# Solid tumors provide niche-specific conditions that lead to preferential growth of *Salmonella*

**DOI:** 10.18632/oncotarget.9071

**Published:** 2016-04-28

**Authors:** Cecilia A. Silva-Valenzuela, Prerak T. Desai, Roberto C. Molina-Quiroz, David Pezoa, Yong Zhang, Steffen Porwollik, Ming Zhao, Robert M. Hoffman, Inés Contreras, Carlos A. Santiviago, Michael McClelland

**Affiliations:** ^1^ Department of Microbiology and Molecular Genetics, University of California, Irvine, CA, USA; ^2^ Departamento de Bioquímica y Biología Molecular, Facultad de Ciencias Químicas y Farmacéuticas, Universidad de Chile, Santiago, Chile; ^3^ Current address: Department of Molecular Biology and Microbiology, Tufts University, Boston, MA, USA; ^4^ Current address: Center for Adaptation Genetics and Drug Resistance, Tufts University, Boston, MA, USA; ^5^ AntiCancer, Inc., San Diego, CA, USA; ^6^ Department of Surgery, University of California, San Diego, CA, USA

**Keywords:** Salmonella Typhimurium, ethanolamine, high-throughput, mammary cancer, 4T1

## Abstract

Therapeutic attenuated strains of *Salmonella* Typhimurium target and eradicate tumors in mouse models. However, the mechanism of *S*. Typhimurium for tumor targeting is still poorly understood. We performed a high-throughput screening of single-gene deletion mutants of *S*. Typhimurium in an orthotopic, syngeneic murine mammary model of breast cancer. The mutants under selection in this system were classified into functional categories to identify bacterial processes involved in *Salmonella* accumulation within tumors. Niche-specific genes involved in preferential tumor colonization were identified and exemplars were confirmed by competitive infection assays. Our results show that the chemotaxis gene *cheY* and the motility genes *motAB* confer an advantage for colonization of *Salmonella* within orthotopic syngeneic breast tumors. In addition, *eutC*, a gene belonging to the ethanolamine metabolic pathway, also confers an advantage for *Salmonella* within tumors, perhaps by exploiting either ethanolamine or an alternative nutrient in the inflamed tumor environment.

## INTRODUCTION

Cancer is one of the leading causes of death worldwide. Many anti-cancer drugs suffer from toxicity and a lack of specificity, affecting healthy tissue and quality of life of the patient. Moreover, when used in advanced stages of disease, conventional treatments often fail, leading to the death of the patient. One barrier to the delivery of conventional anti-cancer drugs is the irregular organization of blood vessels within the tumor tissue that often leads to the development of hypoxic and/or necrotic regions [1]. Interestingly, this niche has also been shown to be favorable for the growth of obligate and facultative anaerobic bacteria [2, 3], which could be exploited for the development of new anti-tumor therapies based on attenuated bacterial vectors.

The most extensive work in this area has been conducted using *Salmonella* [4], due to its ability to grow in the absence and presence of oxygen, and because of the vast knowledge and genetic tools developed in this bacterial model [5]. These are features that provide a potential advantage to *Salmonella* when compared to *Clostridium* and other therapeutic bacteria [2, 6].

Due to limited accessibility, necrotic/hypoxic areas in the tumor tissue are major barriers to many currently available anticancer treatments [3, 7]. The high bacterial selectivity for precisely these areas could lead to an increased anti-tumor efficacy. In addition, bacteria can be used to deliver therapeutics [8]. These therapeutics could themselves be tissue-selective based on unique properties of normal and tumor tissues [9, 10]. Furthermore, after tumor regression, bacteria can be removed by the use of suitable antibiotics. Thus, the presence of bacteria and the therapeutics they deliver can be tightly controlled. Moreover, bacteria can be easily grown, stored, and handled, facilitating the implementation of therapeutic strains [11].

*Salmonella enterica* serovar Typhimurium (*S*. Typhimurium) accumulates preferentially in a wide variety of solid tumors versus normal mouse tissue at a ratio of 1,000:1 [12], seemingly preferring the tumor environment to any other niche in the host. To date, some aspects of the dynamics of solid tumor colonization by *Salmonella* have been described. *Salmonella* resides extracellularly in the hypoxic and necrotic areas within the tumor tissue [13, 14], where it forms biofilms [15]. In addition, using an *in vitro* cylindroid tumor model, it has been shown that *Salmonella* would need chemotaxis and motility associated genes to colonize and use nutrients present in the tumor tissue [16, 17]. After tumor penetration, the bacterial infection leads to a high infiltration of immune cells (mainly neutrophils and dendritic cells) [18], that locate between the viable and necrotic areas of the tumor, limiting the spatial distribution of the bacteria [19].

Some avirulent strains of *S.* Typhimurium retain specificity for tumor tissue colonization, and several attenuated mutants have been used for anti-cancer experiments in animal models [4] and human clinical trials [20, 21]. Attenuated *S.* Typhimurium strains have proved to be very successful in animal models. The auxotrophic strain A1-R has been highly effective against cancers of the prostate [22, 23], breast [24–26], lung [27, 28], pancreas [29–33], ovaries [34, 35] stomach [36], and cervix [37], as well as sarcoma [38–40] and glioma [41, 42], all of which are highly aggressive tumor models.

In the present work, we aimed to identify the bacterial mechanisms involved in the preferential colonization and proliferation of *S.* Typhimurium in an orthotopic, syngeneic murine mammary tumor model. This work builds on our previous efforts indicating that disrupting purine metabolism greatly diminished the ability of *Salmonella* strains to compete in the tumor [43]. The only strain that has been tried in human melanomas, VNP20009, is defective in purine metabolism [20], and melanomas are known to have decreased *de novo* biosynthesis of purine during their invasive stage [44]. Together, these facts may explain why this *Salmonella* strain only colonized some tumor types [21] and failed to eradicate melanomas in humans [20]. This example emphasizes the importance of understanding how bacteria accumulate in tumors for the process of identifying efficient therapeutic strains that can be used in human medicine.

## RESULTS

### Identification of *S*. Typhimurium genes involved in preferential colonization and survival in mammary tumor tissue in BALB/c mice

First, we evaluated the ability of the wild-type *S*. Typhimurium 14028s strain to preferentially colonize and proliferate within mammary tumor tissue of tumor-bearing BALB/c mice. We chose the 4T1 tumor in syngeneic BALB/c mice because our previous work in this mouse model (both with 4T1 and other tumor types) showed that an avirulent derivative of our *Salmonella* strain leads to complete tumor regression, even after metastasis [24, 26, 45].

To induce mammary tumors in BALB/c mice, ~10^6^ 4T1 tumor cells were injected orthotopically into the third mammary gland. Fully developed tumors of ~1 cm diameter (~1 g of weight) were obtained after 7-10 days. Mice were injected intraperitoneally (IP) with ~10^5^ CFU. After 2 days of infection, *S*. Typhimurium was found at a ratio of 100:1 to 1,000:1 in tumor versus the normal sites of accumulation in spleen and liver (data not shown). To identify genes involved in the preferential colonization and proliferation of *Salmonella* within solid tumor tissue, BALB/c mice bearing mammary tumors were injected IP or intratumorally (IT) with ~6×10^6^ CFU of a single-gene deletion mutant library carrying 3,690 mutants in genes of *S*. Typhimurium that were replaced by a kanamycin-resistance cassette (SGD-K) by allelic exchange [46, 47]. After 2 days of infection, less fit mutants were identified based on their relative abundance, as measured by microarray hybridizations [46, 48, 49]. Data obtained from tumor colonization experiments was compared to previously identified genes involved in systemic colonization of BALB/c mice using the same SGD-K library [48], revealing which mutants were at a disadvantage relative to wild type in the tumor environment. Examples of candidate mutants under negative and positive selection in tumors but not in spleen are presented in Supplementary Tables S1 and S2.

**Figure 1:**
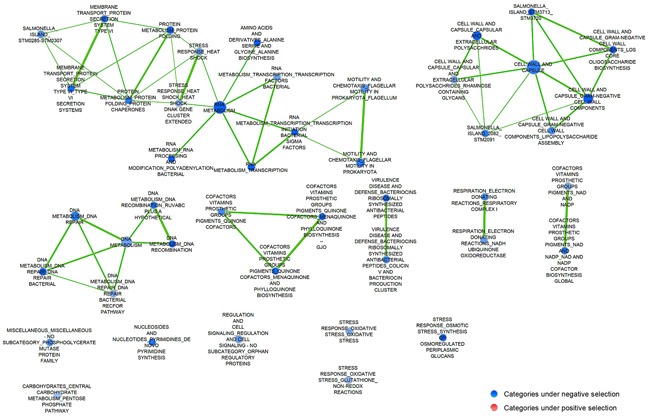
Pathways enriched for genes under selection during growth in tumor A ranked list of all mutants, based on their Limma *t* scores for selection relative to wild type, was used as an input for Gene Set Enrichment Analysis [50] (http://www.broadinstitute.org/gsea/index.jsp). A network of gene sets enriched at a false discovery rate (FDR) < 25% was plotted using cytoscape [82]. Circle size is proportionaly to the number of genes corresponding to mutants under selection in each data set. The thickness of the connecting lines indicates the number of significantly selected genes shared by related ontologic categories. Blue nodes represent gene sets with a significant number of genes where the corresponding mutants confer a growth disadvantage relative to wild-type bacteria.

We performed functional category enrichment analyses [50] of the genes corresponding to the mutants under selection to identify bacterial processes involved in colonization and survival within tumors (Figure 1) and healthy organs (Supplementary Figure S1). Several pathways or functional categories (defined in Supplementary Table S3) had a stronger selection in the tumor environment when compared to healthy tissue, such as protein transport, DNA and RNA metabolism, chemotaxis and motility, and cell wall and capsule components. It is noteworthy that mutants in pathogenesis-related genes (e.g., SPI-1, SPI-2 and secreted effectors), and genes associated with the Lipopolysaccharide (LPS) of the outer membrane, are negatively selected in spleen and liver of regular BALB/c mice [48] when compared to tumor tissue (compare Figure 1 and Supplementary Figure S1). This suggests that many systems involving pathogenesis-associated genes are less important in the tumor niche. Most bacteria in spleen and liver are inside host cells involved in innate immunity, particularly macrophages [51, 52]. As we will show below, *Salmonella* are generally outside host cells within tumors.

### The colonization of the solid tumor tissue is an active mechanism

The fact that motility and chemotaxis-linked functional categories were important for colonization of tumors and not healthy organs (compare Figure 1 and Supplementary Figure S1), suggests an active mechanism of tumor penetration by this pathogen. This is in agreement with previous work on motile attenuated strains of *Salmonella* and non-motile strains of *Escherichia coli* [53–55]. To confirm this, a mutant in *cheY*, which encodes the response regulator of the two-component system that controls chemotaxis in several bacterial species [56], was assessed for its ability to colonize spleen, liver and tumors. We first (a) confirmed RNA expression of the wild-type gene in *Salmonella* within tumors, and (b) transduced the mutation to the wild type background, to reduce the possibility of second site mutations. In addition, (c) the initial screen of all mutants in mice involved two antibiotic cassettes in the opposite orientation to reduce the possibility of polarity [47].

**Figure 2:**
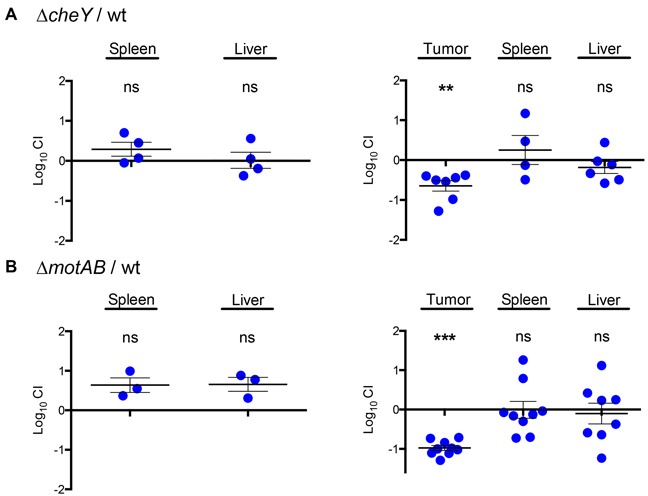
The chemotaxis and motility genes *cheY* and *motAB* confer an advantage during colonization of solid tumor tissue *in vivo* A 1:1 ratio of either the Δ*cheY* **A.** or Δ*motAB* **B.** mutant to wild-type *S*. Typhimurium 14028s was inoculated IP in regular (left) or tumor-bearing (right) BALB/c mice. After 48 hours, bacteria were recovered from tissue and competitive index (CI) values were calculated as mean ratios of mutant to wild type, normalized to the input ratio and converted logarithmically. Thus, a value of zero indicates that the two strains in comparison are equally fit, a value < 0 indicates that the tested strain was outcompeted by the wild type and a value > 0 indicates that the tested strain outcompeted the wild type. Error bars denote standard error. Statistical significance was determined using a two-tailed Student's *t* test (** P < 0.005, *** P < 0.001).

We then performed competitive assays by IP inoculation with ~10^6^ CFU of a 1:1 mixture of the mutant and the wild-type strain in either normal or tumor-bearing BALB/c mice. Two days after infection, organs were collected and the number of wild type and mutant bacteria was obtained to determine the competitive index (CI). Our results show that the Δ*cheY* mutant had an equal ability to colonize spleens and livers of regular BALB/c mice (Figure 2A, left panel), suggesting that the *cheY* gene is not required for colonization after systemic delivery. In contrast, when the Δ*cheY* mutant was subjected to selection in tumor-bearing mice, the strain was outcompeted by the wild type in tumor tissue. On the other hand, the mutant retained the same ability as wild type to colonize the liver and spleen in these mice (Figure 2A, right panel). Thus, our competitive assay allowed the identification of niche-specific genes involved in the process of preferential tumor colonization within the same animal after IP delivery.

To confirm that the chemotaxis-driven tumor penetration is an active mechanism of *S*. Typhimurium, we generated a Δ*motAB* mutant in which the flagellar motor [57] is inactive but all the chemotaxis and flagella genes are intact. When the Δ*motAB* mutant was subjected to *in vivo* competition against the wild-type strain, it had similar systemic colonization abilities in normal BALB/c mice (Figure 2B, left panel). However, this mutant had an impaired colonization in tumor tissue when compared to the wild-type strain (Figure 2B, right panel), just like the Δ*cheY* mutant. In addition, we confirmed that the *motAB* region is transcribed by wild-type *Salmonella* in tumors (data not shown), as in the case of *cheY*.

### The solid tumor tissue environment provides niche-specific nutrients that lead to the preferential growth of *Salmonella.*

The intestinal phase of infection by *S*. Typhimurium includes internalization in epithelial intestinal cells mediated by the action of a type III protein secretion system (T3SS) encoded in the *Salmonella* Pathogenicity Island 1 (SPI-1). This system injects effector proteins that induce a rearrangement of the actin cytoskeleton and promote the formation of membrane ruffles and bacterial engulfment in a process similar to phagocytosis [58, 59]. Then, survival of *Salmonella* within host cells is facilitated by a second T3SS encoded in SPI-2 and its corresponding effector proteins. The invasion process triggers the production of the pro-inflammatory cytokines IL-8, IL-1β and IL-18 that recruit immune cells to the site of infection leading to inflammation [60].

In the inflamed gut model, where *Salmonella* needs to invade epithelial cells for a successful infection, it has been shown that the inflammation caused by infiltrating neutrophils provides alternative carbon, nitrogen and/or electron acceptors that allow *Salmonella* to outcompete the resident microbiota and proliferate within the intestinal lumen [61–64]. Similarly, it has been shown that the center of solid tumors is typically inflamed and relatively anaerobic [65, 66]. Moreover, a high infiltration of immune cells (neutrophils, dendritic cells) occurs after bacterial infection [18, 19], limiting the *Salmonella* location within tumor tissue to the necrotic areas which are less accessible to these immune cells. Extensive cell death in these areas of the tumor may provide alternative carbon sources such as ethanolamine, a component of cell membranes, creating a favorable niche for the growth of this pathogen as is also seen in the inflamed gut [61, 67, 68].

**Figure 3:**
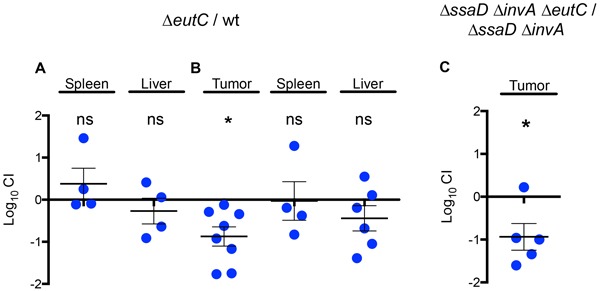
A gene in the ethanolamine degradation cluster confers an advantage for *Salmonella* growth in the tumor environment that does not require SPI-1 mediated host cell invasion A. 1:1 ratio of the Δ*eutC* mutant to wild-type *S.* Typhimurium 14028s was inoculated IP in regular **A.** or tumor- bearing **B.** BALB/c mice. **C.** A 1:1 ratio of a Δ*ssaD* Δ*invA* Δ*eutC* mutant and a Δ*ssaD* Δ*invA* mutant was inoculated IT in tumor-bearing BALB/c mice. After 48 hours, bacteria were recovered from the designated tissues and CI values were calculated as mean ratios of the two mutants, normalized to the input ratio and converted logarithmically. A value of zero indicates that the two strains in comparison are equally fit, a value < 0 indicates that the tested strain was outcompeted by the wild type and a value > 0 indicates that the tested strain outcompeted the wild type. Error bars denote standard error. Statistical significance was determined using a two-tailed Student's *t* test (* P < 0.05).

To test our hypothesis, we chose the *eutC* gene which encodes the small subunit of the ethanolamine ammonia-lyase enzyme that catalyzes the formation of acetaldehyde from ethanolamine, the first step in the ethanolamine utilization pathway [61]. Therefore, the lack of *eutC* will impair the entire ethanolamine metabolic route. We first confirmed that *eutC* RNA is transcribed in wild-type *Salmonella* inside tumors (data not shown), and then generated a Δ*eutC* mutant and tested its ability to colonize the spleen and liver of normal mice and tumors from tumor-bearing BALB/c mice in competitive assays. Mice were inoculated IP with ~10^6^ CFU of a 1:1 mixture of the Δ*eutC* mutant and the wild-type strain to confirm this phenotype (Figure 3A). When the Δ*eutC* mutant was inoculated IP in tumor-bearing mice, it showed an impaired colonization only for tumor tissue and not for liver or spleen. As in the case of the Δ*cheY* and Δ*motAB* mutants, the Δ*eutC* mutant colonized spleen and liver of regular BALB/c mice at wild-type levels (Figure 3B). Our results indicate that the *eutC* gene is not required for systemic colonization in regular BALB/c mice.

### Invasion of tumor cells is not necessary for *eutC* utilization

Evidence from our screen indicated that mutants in genes involved in invasion of epithelial cells and the cells of the innate immune system (i.e., SPI-1 and SPI-2) were not under selection in tumors. We used a gentamicin protection assay to quantitate the number of bacteria protected by being inside host cells (Supplementary Figure S2). The protected fraction was very low at 1 h after IT delivery and after 2 days of IP delivery. In a preliminary FACS analysis, the few protected wild-type bacteria were in immune system cells (unpublished results).

To test if *Salmonella* invasion of any cell type is required for the use of *eutC* in the tumor tissue, we generated a strain carrying deletions of *invA* and *ssaD* [64], which encode structural components of the T3SS encoded in SPI-1 and SPI-2, respectively. We compared the survival of the non-invasive mutants Δ*invA* Δ*ssaD* and Δ*invA* Δ*ssaD* Δ*eutC* in tumor tissue by IT injection of ~10^8^ CFU of a 1:1 mixture of both strains to determine the CI. The Δ*invA* Δ*ssaD* Δ*eutC* presented an impaired growth within tumor tissue when compared to the Δ*invA* Δ*ssaD* mutant (Figure 3C), indicating that the *eutC* gene is required for *Salmonella* survival in the tumor tissue even when invasion of tumor cells is not a component of the inflammatory response. Therefore, our results suggest that the use of a metabolic route involving the EutC protein is independent of invasion of tumor cells.

## DISCUSSION

*S.* Typhimurium accumulates preferentially in tumor tissue versus normal tissue in mice [12]. Attenuated *S*. Typhimurium strains have been frequently used in tumor therapies, which have proved to be very successful in animal models but not when used in human clinical trials [20, 21].

One of the reasons for the lack of effectiveness of particular strains in humans when compared to the success achieved in animal models could be due to the use of auxotroph bacterial mutants that may display differentially impaired abilities to colonize the tumor tissues in these hosts. Mutants that accumulate to an ideal level, with great safety, may be developed, but without knowing how the bacterium reacts to the tumor environment it is difficult to pinpoint mutants with a higher effectiveness in humans. Many avirulent mutants, such as most amino acid auxotrophs [2, 13], have yet to be tried. Once we understand how the bacterium reacts to the tumor environment at a molecular level, it will be easier to identify candidate metabolic genes that can be knocked out without affecting bacterial tumor-regressive capabilities.

To establish the mechanism by which *Salmonella* preferentially colonizes tumor tissue, we used a negative selection strategy employing a single-gene deletion mutant library, SGD-K [46–48, 69]. This strategy allowed us to identify bacterial processes that are needed for fitness in tumors of mice. Mutants in genes needed to reach the tumor tissue were negatively selected in our genetic screens (Supplementary Table S1 and Figure 1). Those mutants were then compared to genes required for systemic colonization that had been identified using the same mutant library (Supplementary Figure 1S), where some, such as genes required for motility and chemotaxis, were found to be dispensable.

Conflicting data exist in the literature regarding the molecular mechanism of tumor colonization by *Salmonella*. It has been proposed that flagellar or active motor function leading to chemotaxis is essential for bacterial accumulation and proliferation in an *in vitro* tumor cylindroid model [16, 17]. Our data supports that conclusion. In contrast, a passive mechanism has been suggested in work by others on CT26 murine colon carcinoma tumors in BALB/c mice, where an increase of TNF-α in the blood after intravenous administration of a *hisG aroA* double mutant of *S*. Typhimurium promoted a blood influx and therefore, bacteria were able to reach this tissue in a passive mechanism [70]. In this model, neither *cheY* nor flagellar genes were needed for tumor colonization. However, an *aroA* mutant exhibit among the most severe reduced fitness of *Salmonella* in tumors [43], so this passive mechanism may only be relevant for bacteria that are less fit for accumulation in tumors, or may be highly dependent on the inoculation route. In another study, chemotaxis genes were not needed for colonization of tumor tissue but were needed for full colonization of deep organs (spleen, liver and lung) of BALB/c mice bearing subcutaneous xenografts of human prostate and breast cancer cells [43]. Nevertheless, the authors describe flagellar genes as needed for tumor colonization, which could imply an active mechanism for *Salmonella* to reach tumor tissue *in vivo.*

Given that chemotaxis and motility genes were identified as needed for accumulation in tumor tissue and not in normal tissues in our screen, and this difference was confirmed thereafter by competitive assays in our *in vivo* animal model, we can suggest an active mechanism of *Salmonella* to penetrate and survive within tumor tissue *in vivo*. We propose that *Salmonella* penetrates this tissue by sensing nutrients and then establishes a niche, perhaps partially protected from parts of the immune system, in which it survives by the action of its metabolism and transport mechanisms. The differences found in the literature indicate the importance of comparing different routes of infection, the use of syngeneic, orthotopic tumor models and bacterial strains with a variety of different attenuations. For example, it has been shown that mutants of genes belonging to SPI-13 have a reduced fitness in normal tissues and unchanged fitness in tumors, when delivered IT [43]. However, in our analyses (Figure 1, and data not shown), mutants belonging to this gene cluster are also negatively selected in tumor when injected IP. These differences are important because intratumoral treatments are (often) impossible for deep tumors and metastases of unknown location.

According to our data, *eutC*, the gene encoding the small subunit of the enzyme responsible for the first step in the ethanolamine utilization pathway, is important for *Salmonella* proliferation within tumor tissue. The *eut* gene cluster has been implicated in the utilization of ethanolamine present in the gut as a nitrogen source by enterohemorrhagic *Escherichia coli* (EHEC) and *Salmonella*. Noteworthy, ethanolamine is not used by commensal microbiota and helps EHEC or *Salmonella* to get an advantage for growth [61, 71]. Moreover, *S*. Typhimurium uses ethanolamine for signaling to recognize and adapt to different niches within the host, leading to successful intestinal infection and intramacrophage survival [72]. We speculate that *Salmonella* could be using the ethanolamine from cell membranes abundantly present due to cell death and inflammation damage within tumor tissue. Alternatively, a similar unknown substrate that is metabolized with the help of EutC may be used as food source.

Since *Salmonella* is a facultative intracellular pathogen able to trigger inflammation via invasion of mammalian cells, we tested the intratumoral survival of a *eutC* mutant with and without the ability to invade host cells, by knocking out the T3SSs encoded in SPI-1 and SPI-2. The *eutC* mutant had an impaired growth relative to the wild type within tumor tissue after IP (data not shown) or IT delivery, regardless of the presence or absence of both T3SSs. These results suggest that inflammation via innate immunity, independent from cell invasion, augments the available amount of a EutC substrate in tumor tissues, leading to the preferential colonization and proliferation by *Salmonella*. Going forward, further characterization of invasion and tumor regression using mice with impairments in various parts of the immune/inflammatory response will be of immense interest. The fact that the *cheY*, *motAB* and *eutC* mutants colonized spleen and liver of tumor-bearing mice normally and only had impaired growth within tumor tissue suggests that the tumor environment provides niche-specific features that result in the preferential proliferation of *Salmonella.*

Once established, *Salmonella* may exert its anti-tumor effect by a number of mechanisms, ranging from simple competition for nutrients and oxygen to immune responses. Just a few of those proposed mechanisms include induction of apoptosis of tumor cells [14], and/or a bystander effect of cross-presentation of bacterial antigens by dendritic cells present in the tumor environment [18], thus recruiting cytotoxic T cells that will ultimately lead to tumor killing. *Salmonella* reduces the capacity of tumor myeloid suppressor cells, thus enhancing the host's anti-tumor immune response [73]. Activation of TLR5 by *Salmonella* flagellin stimulates cytotoxic lymphocyte-mediated tumor immunity [74]. We have shown that delivery of αPD-L1 and αCTLA-4 via *Salmonella* can help to rescue dysfunctional endogenous tumor-specific CD8(+) T cells and eradicate advanced immunogenic tumors [75]. *Salmonella* immunotherapy for B-cell lymphoma induces broad anti-tumor immunity with therapeutic effect. In another potential mechanism, cancer cells arrested in G_0_/G_1_ (which are usually the majority of cancer cells in a tumor [76]), are induced by *Salmonella* to attempt the re-start of the cell cycle. This leads to cancer cell death, possibly due to unbalanced DNA replication [36].

Taken together, our data support a model in which *Salmonella* actively penetrates and/or actively remains in tumors, primarily outside tumor cells, where the bacterium senses nutrients that may include some of those that the bacterium also exploits when it is growing extracellularly in the gut.

The knowledge generated by this work is a step towards the construction of safer and potentially more effective tumor-targeted therapies using attenuated strains of *Salmonella*.

## MATERIALS AND METHODS

### Bacterial strains, media and growth conditions

Wild-type strain 14028s was obtained from ATCC. Bacteria were routinely grown in Luria-Bertani (LB) medium (10 g/l tryptone, 5 g/l yeast extract, 5 g/l NaCl) at 37°C with aeration. When required, LB medium was supplemented with chloramphenicol (Cam; 20 mg/l) or kanamycin (Kan; 50 mg/l). Media were solidified by the addition of agar (15 g/l). Strains used are listed in Supplementary Table S4.

### Standard DNA techniques

Mutant strains with specific allelic replacement of the *motAB* gene cluster or the *cheY*, *eutC*, *ssaD*, *invA* and *ssaD* genes with a Kan or Cam-resistance cassette were constructed using the Lambda-Red recombination method, also referred to as the “Red-swap” method, using either plasmid pCLF2 (GenBank accession number HM047089) or pCLF4 (GenBank accession number EU629214) as template [46, 47, 77]. Primers for PCR amplification were designed based on the reported sequence of *S*. Typhimurium 14028s (Supplementary Table S5). Correct allelic replacement was confirmed by PCR using the gene-specific out5 and pCLF4(P1)Bam primers for each mutant. When required, PCR products were purified using the QIAquick PCR Purification Kit (QIAGEN) as recommended by the manufacturer, and eluted with nuclease-free water. The selected mutations were transduced to the wild-type background using phage P22 HT105/1 *int*-201.

### Cell culture conditions

The murine mammary tumor cell line 4T1 expressing the red fluorescent protein (4T1-RFP, Anticancer, Inc) was routinely maintained in RPMI-1640 medium (25 mM HEPES, L-Glutamine) supplemented with 10% of inactivated bovine fetal serum (FBS) at 37°C in a 5% CO_2_ atmosphere.

### Tumor-bearing mice colonization experiments

All mouse studies were conducted at AntiCancer, Inc. (San Diego, CA) with an Institutional Animal Care and Use Committee (IACUC) protocol specifically approved for this study and in accordance with the principals and procedures outlined in the National Institute of Health Guide for the Care and Use of Animals under Assurance Number A3873-1.

Tumors were generated by the orthotopic injection of 1×10^6^ murine mammary tumor 4T1-RFP cells at the third mammary gland. After 7-10 days, fully developed tumors were obtained and colonization studies were performed. To study colonization by wild type *S*. Typhimurium 14028s, ~10^5^ CFU were injected IP into groups of six BALB/c mice (6-10 week-old female). Mice were sacrificed 2 days post infection; tumor, spleen and liver were removed and homogenized and CFU counts were obtained.

For negative selection studies using the SGD-K mutant library, a frozen glycerol aliquot of the library was used to inoculate 25 ml of LB Kan. After overnight growth with constant aeration at 37°C, bacteria were centrifuged and cell pellets were washed with sterile PBS, diluted and plated for titer on LB Kan agar plates. A group of six BALB/c mice (6-10 week-old female) was infected IP or IT with ~6×10^6^ CFU of the library. Samples of the inoculated material (*input library*) were kept frozen until further use. Mice were sacrificed 2 days post infection; organs were removed and homogenized in 5 ml of sterile PBS to determine CFU counts. An aliquot of 200 μl from each homogenate was used for serial dilution and titer on LB Kan plates. The remaining homogenate was inoculated into 25 ml of LB Kan for overnight growth with agitation at 37°C. The bacteria obtained from the homogenized organs (*output library*) were pelleted, washed with sterile PBS and stored frozen until further use.

### Evaluation of *in vivo* tumor cell invasion by *Salmonella*

To assay *in vivo* invasion, gentamicin protection assays were performed. Tumor-bearing mice were injected IT with 20 μl of PBS containing 10^8^ CFU, or IP with 100 μl of PBS containing 10^5^ CFU of the *S*. Typhimurium 14028s wild-type strain or the non-invasive derivative *S*. Typhimurium 14028s DΔSPI-1. Invasion was performed for 1 h (IT injection) or 2 days (IP injection). After invasion, mice were sacrificed, tumors were extracted, minced and incubated for 90 min in RPMI supplemented with 1 mg/ml of collagenase/dispase (Roche Diagnostics), 0.3 mg/ml Liberase TL (Roche Diagnostics), 0.125% of DNAse I and gentamicin (200 μg/ml) in order to generate a single cell suspension and kill extracellular bacteria. Next, cells were washed and lysed with 0.5% of sodium deoxycholate (DOC) in PBS. Serial dilutions were prepared and plated on LB agar plates to determine viable intracellular bacteria. The percentage of bacterial invasion was calculated according to the following equations:

$$\begin{array}{l} \text{Invasion }\%\;(\text{IT})=(\text{CFU recovered}*100)/\text{CFU inoculums}\\ \text{Invasion }\%\;(\text{IP})=(\text{CFU recovered}*100)/(\text{CFU recovered from tumor tissue}) \end{array}$$

### Labeling of DNA adjacent to mutant insertions and microarray analysis

The DNA adjacent to the deletion in each mutant from the input and output libraries was specifically amplified as described [46, 48], with modifications. Briefly, genomic DNA was extracted (GenElute Bacterial Genomic DNA kit - Sigma-Aldrich) and sonicated. Then, polyA tails were added to the DNA fragments using terminal transferase (TdT) following the instructions of the manufacturer. Nested PCR was used to amplify the polyA-tailed fragments containing the Kan amycin resistance cassette carrying the P_T7_ and the genomic DNA downstream the insertion. Primers used are listed in Supplementary Table S5. An aliquot of the nested PCR reaction was used for *in vitro* transcription (AmpliScribe T7 transcription kit - Epicentre), following the manufacturer's protocol. During this process, Cy5-UTP or Cy3-UTP was incorporated to generate labeled RNA. The remnant DNA was digested with RNase-free DNase (Epicentre) and the RNA was purified (RNeasy Mini Kit - QIAGEN) and used for hybridization.

### Hybridization and data analysis

A total of 4 μg of labeled RNA were hybridized onto high-density Nimblegen oligonucleotide tiling arrays (387.000 oligos) [48]. After incubation, slides were washed and scanned using the Axon GenePix 4000B laser scanner with GenePix Pro 6.0 software. Fluorescence signal intensities were quantified using NimbleScan 2.4 software (Roche Diagnostics). Intensities for five probes following the 3′ insertion site for each mutant were extracted using custom Perl scripts. These data were background-corrected and quantile normalized in Bioconductor. Normalized intensities for the five probes were summarized by calculating the median, which was used in further statistical analysis. Limma [78] was used to calculate fold-changes and false-discovery rates (FDR) associated with each mutant, between the output and input samples.

GSEA (V2.2.0) [50] was used to identify pathways that were enriched in genes that were needed (mutants under negative selection) or genes that were deleterious in this environment (mutants under positive selection) during growth in tumor. A ranked list of all genes based on their Limma *t* score (indicating significance of observed differences) was used as an input for GSEA. Gene sets for metabolic and signaling pathways were constructed based on SEED annotations [79]. Gene sets for virulence factors and genomic islands were constructed based on the virulence factor database VFDB [80] and manual curation [81]. A network of gene sets enriched at a false discovery rate (FDR) < 25% was plotted using cytoscape [82].

### Phenotype confirmation by competitive assays

For phenotype confirmation of individual mutants, competition assays were performed. Groups of 3-5 BALB/c mice (6-10 week-old female) were inoculated IP with ~10^6^ CFU of a mixture containing a defined mutant and the wild-type *S*. Typhimurium 14028s in a 1:1 ratio to determine the competitive index. Mice were euthanized two days post infection and CFU counts from tumor, spleens and livers were obtained. CI values were calculated as a mean ratio of mutant to wild type, normalized to the input ratio and converted logarithmically using the following formula: Log_10_ ((mutant output/wild-type output)/(mutant input/wild-type input))

## SUPPLEMENTARY FIGURES AND TABLES








