# Supplementary material for: Solid tumors provide niche-specific conditions that lead to preferential growth of Salmonella
Source: Oncotarget. 2016 Apr 28;7(23):35169–80. doi: 10.18632/oncotarget.9071 (PMC5085218; doi:10.18632/oncotarget.9071)
Supplement: Supplementary file 1 [file oncotarget-07-35169-s001.pdf]

SUPPLEMENTARY FIGURES AND TABLES

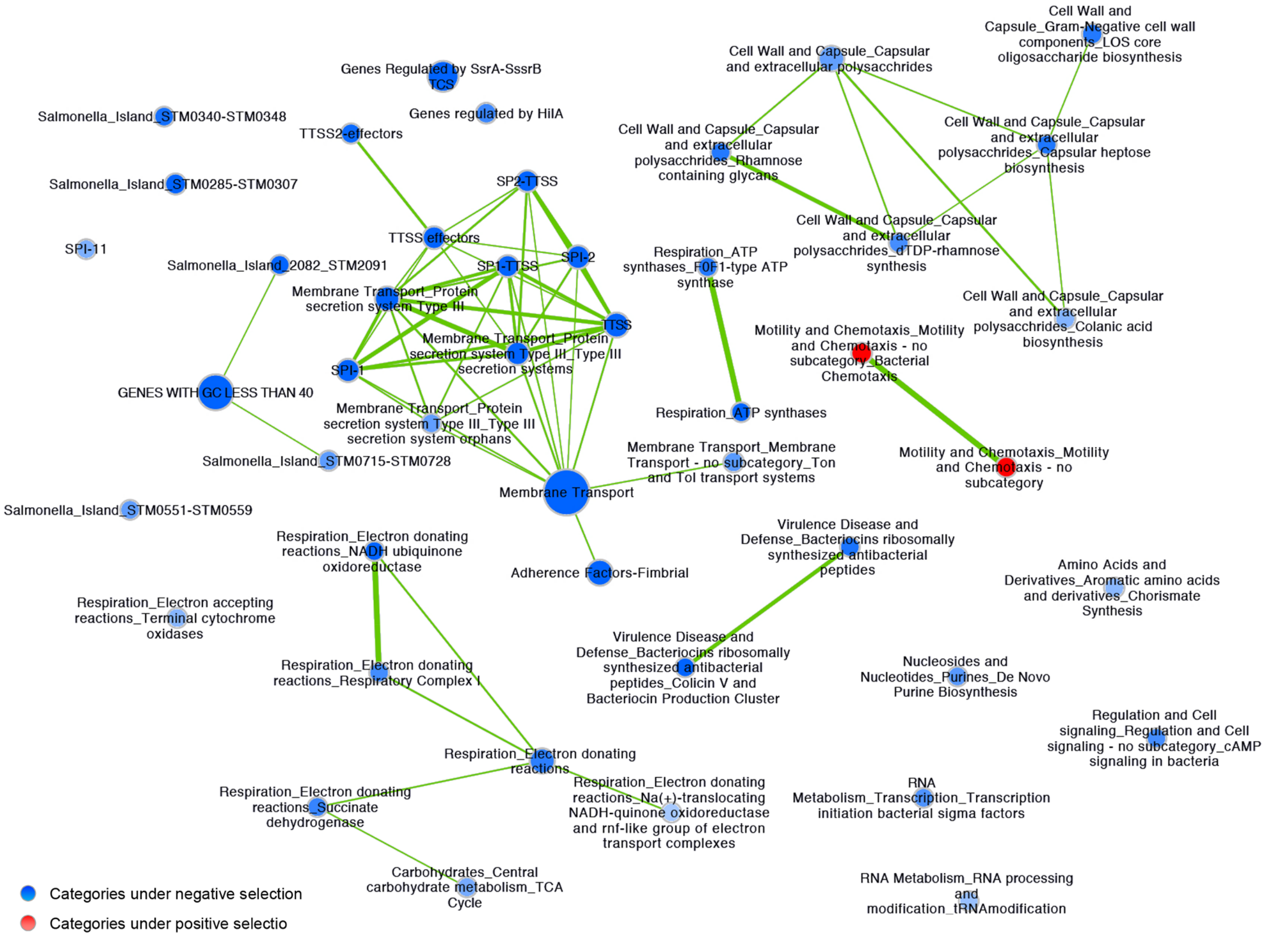

Supplementary Figure S1: Pathways enriched for genes under selection during growth in spleen. A ranked list of data for all *S.* Typhimurium mutants, based on their Limma t scores for selection in spleen of BALB/c mice relative to wild type, was used as an input for Gene Set Enrichment Analysis [50] (http://www.broadinstitute.org/gsea/index.jsp). A network of gene sets enriched at an FDR < 25% was plotted using cytoscape [82]. Circle size is proportional to the number of genes corresponding to mutants under selection in each category. The thickness of the connecting lines indicates the number of significantly selected genes shared by related ontologic categories. Blue nodes represent gene sets with a significant number of genes where the corresponding mutants confer a growth disadvantage relative to wild-type bacteria. Red nodes represent gene sets with a significant number of genes where the corresponding mutants confer a growth advantage relative to wild-type bacteria.

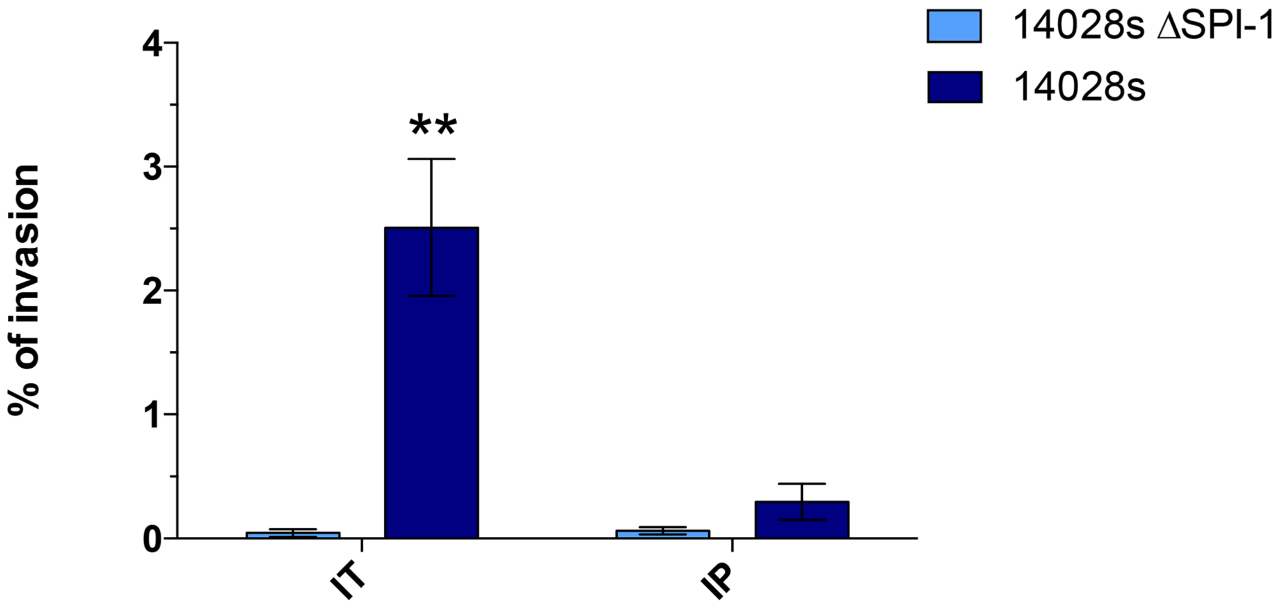

Supplementary Figure S2: Low level of S. Typhimurium invasion of host cells in tumors. Tumor-bearing BALB/c mice were injected IT **A.** or IP **B.** with *S.* Typhimurium wild type (14028s) or a non-invasive mutant (14028s ΔSPI-1). For intratumoral experiments, ~10^8 CFUs of each strain were injected directly into mammary tumor tissue and intracellular CFU counts were evaluated by a gentamicin protection assay 1h after inoculation. Invasion percentage was calculated based on initial inoculum. For assessment of tumor cell invasion after intraperitoneal delivery, ~10^8 CFUs were administered IP and 2 days post infection intracellular CFU counts were evaluated by a gentamicin protection assay. Invasion percentage was calculated based on total CFU recovered per organ after 48 h. Replicates were conducted at least in triplicate and error bars show standard error. Statistical significance was determined by Kruskall-Wallis and Dunn's post-test.

Supplementary Table S1: Mutants less fit in tumors but not less fit in spleen. Data are color coded on an arbitrary scale to represent the direction and amount of change. A partial list of arbitrary cutoff thresholds for generating the list include a log2 fold change of <-0.5 in tumor, T value <-1.4 in tumor, and an FDR <0.5 in tumor. Difference between spleen and tumor T value of >1.4. Genes in yellow are investigated further.

See Supplementary File 1

Supplementary Table S2: Mutants more fit in tumors but not more fit in spleen. Data are color coded on an arbitrary scale to represent the direction and amount of change, A partial list of arbitrary cutoff thresholds for generating the list include a log2 fold change of >0.5 in tumor, T value >1.4 in tumor, and an FDR <0.5 in tumor. Difference between spleen and tumor T value of <-1.4.

See Supplementary File 2

Supplementary Table S3: Pathways and categories used in Gene Set Enrichment Analysis. The *Salmonella* gene names associated with each pathway and category queried for gene set enrichment analysis in Figure 1 and Supplementary Figure S1.

See Supplementary File 3

Supplementary Table S4: Strains used in this study.

Strain	Genotype or relevant phenotype	Source
***S.* Typhimurium**		
14028s	*S.* Typhimurium wild type	Laboratory collection
Δ*cheY*	14028s Δ*cheY*::Kan	Present study
Δ*motAB*	14028s Δ*motAB*::Cam	Present study
Δ*eutC*	14028s Δ*eutC*::Kan	Present study
Δ*invA* Δ*ssaD*	14028s Δ*invA*::Kan Δ*ssaD*::Cam	Present study
Δ*invA* Δ*ssaD* Δ*eutC*	14028s Δ*invA*::Cam Δ*ssaD*::Kan Δ*eutC*::FRT	Present study

Supplementary Table S5: Primers used in this study

Primer	Sequence
**Transposon mutagenesis and amplification for hybridization**	
$CCT_{24}VN$	CCTTTTTTTTTTTTTTTTTTTTTTTTTVN
FRT-Out3_1	TTCCTATACTTTCTAGAGAA
FRT-Out3_2	TAGGAACTTCGGAATAGGAA
**Mutagenesis of *cheY*, *motAB* and *eutC***	
cheY_H1+P1	GTAGTATTTTATGGCGGATAAAGAGCTTAAATTTTTGGTTGTGCAGGCTGGAGCTG CTTC
cheY_H2+P2	CATCGCATCCTCACATGCCCAGTTTCTCAAAGATTTTGTTCATATGAATATCCTCCT TAG
cheY_out5	CCAGTCCGGCAGTGATTATT
motAB_H1+P1	CGGTTATGTCATGACCGGCGGACACCTTGGGGCACTCTATGTGCAGGCTGGAGCT GCTTC
motAB_H2+P2	GCTCACGCTATCACCTCGGTTCCGCTTTTGGCGATGTGGGCATATGAATATCCTCC TTAG
motAB_out5	GCGTTTTGTTGAAAGTGGGT
eutC_H1+P1	GGATAACACCATGGATCAAAAACAGATTGAAGAAATTGTAGTGCAGGCTGGAGC TGCTTC
eutC_H2+P2	TGATGTCTCCTTAACGGGTCATGTTGATGCCGGACGCTTTCATATGAATATCCTCC TTAG
eutC_out5	ACTACGATCCGTTCCTGGTG
invA_H1+P1	GATACCTATAGTGCTGCTTTCTCTACTTAACAGTGCTCGTGTGCAGGCTGGAGCTG CTTC
invA_H2+P2	AATTAAGCCCTTATATTGTTTTTATAACATTCACTGACTTCATATGAATATCCTCCT TAG
invA_out5	TGAGGGTTCGCTATTAACCG
ssaD_H1+P1	GTAGTAAATAATGGCATATCTCATGGTTAATCCAAAGAGTGTGCAGGCTGGAGCTG CTTC
ssaD_H2+P2	CATTTTCCACTCACTTAAAATCTAATGGATAGTTAATCAACATATGAATATCCTCC TTAG
ssaD_out5	CGGTGGTGCTAGTGGTTTTT
pCLF4(P1)Bam	CGGGATCCGGACTGGCTTTCTACGTGTTCC

Sequences aligning with P1 and P2 regions in pCLF2 and pCLF4 are underlined.
