# Supplementary material for: Solid tumors provide niche-specific conditions that lead to preferential growth of Salmonella
Source: Oncotarget. 2016 Apr 28;7(23):35169–80. doi: 10.18632/oncotarget.9071 (PMC5085218; doi:10.18632/oncotarget.9071)
Supplement: Supplementary file 2 [file oncotarget-07-35169-s002.pdf]

Supplementary Table S1. Mutants less fit in tumors but not less fit in spleen.

Data are color coded on an arbitrary scale to represent the direction and amount of change

A partial list of arbitrary cutoff thresholds for generating the list include a log2 fold change of <-0.5 in tumor, T value <-1.4 in tumor, and an FDR <0.5 in tumor. Difference between spleen and tumor T value of >1.4

Genes in yellow are investigated further

Gene order on genome	Gene	14028_gene	Gene_Type	Gene_name	start	end	strand	tumor Log2 Fold change versus input	tumor t value	tumor False Discovery Rate	spleen Log2 Fold change versus input	spleen t value	speen t value minus tumor t value	final_function	RAST Function
6	STM0002	STM14_00	CDS	thrA	337	2799	+	-4.23	-3.49	0.09	1.14	1.14	4.63	bifunctional aspartokinase I/homeserine dehydrogenase I; multifun	Aspartokinase (EC 2.7.2.4) / Homoserine dehydrogenase (EC 1.1.1.3)
7	STM0003	STM14_00	CDS	thrB	2801	3730	+	-1.02	-2.19	0.28	1.23	1.97	4.16	homoserine kinase; catalyzes the formation of O-phospho-L-homos	Homoserine kinase (EC 2.7.1.39)
12	STM0008	STM14_00	CDS	mogA	8729	9319	+	-2.55	-5.06	0.02	1.51	2.24	7.30	molybdenum cofactor biosynthesis protein; forms a trimer; related	Molybdopterin biosynthesis molybdochelatase MogA
16	STM0013	STM14_00	CDS	dnaJ	13595	14734	+	-2.45	-5.18	0.02	0.40	0.78	5.95	chaperone protein DnaJ; chaperone Hsp40; co-chaperone with Dna	Chaperone protein DnaJ
21	STM0018	STM14_00	CDS		17867	19966	+	-1.39	-2.17	0.28	-0.26	-0.21	1.96	putative exochitinase	Chitinase (EC 3.2.1.14)
30	STM0027	STM14_00	CDS	bcfG	30478	31209	+	-1.02	-2.79	0.17	-0.37	-0.57	2.21	fimbrial chaparone; fimbrial chaperone (gi|4959517)	Hypothetical fimbrial chaperone ycbF precursor
46	STM0042	STM14_00	CDS	xynT [R]	50451	51824	-	-2.63	-2.39	0.23	0.66	1.56	3.94	putative sodium galactoside symporter; similar to Escherichia coli p	Xyloside transporter XynT
67	STM0066	STM14_00	CDS	carA	75881	77029	+	-5.49	-5.77	0.01	0.80	0.69	6.46	carbamoyl phosphate synthase small subunit; catalyzes production	Carbamoyl-phosphate synthase small chain (EC 6.3.5.5)
77	STM0075	STM14_00	CDS	fixA	89396	90166	+	-1.47	-2.30	0.25	-0.18	-0.30	2.00	putative electron transfer flavoprotein FixA; required for anaerobic	Electron transfer flavoprotein, beta subunit FixA
78	STM0076	STM14_00	CDS	fixB	90182	91123	+	-1.62	-3.37	0.10	-0.18	-0.30	3.07	putative electron transfer flavoprotein FixB; involved in electron tra	Electron transfer flavoprotein, alpha subunit FixB
79	STM0077	STM14_00	CDS	fixC_1	91173	92459	+	-1.06	-2.28	0.25	-0.40	-0.46	1.82	putative oxidoreductase FixC; FAD/NAD(P)-binding domain; possibl	Probable electron transfer flavoprotein-quinone oxidoreductase FixC (EC
91	STM0089	STM14_01	CDS	apaG	102995	103372	-	-0.64	-1.64	0.44	0.24	0.33	1.97	ApaG; protein associated with Co2+ and Mg2+ efflux	ApaG protein
115	STM0112	STM14_01	CDS	leuB	131788	132879	-	-1.49	-2.74	0.17	0.14	0.33	3.07	3-isopropylmalate dehydrogenase; catalyzes the oxidation of 3-isop	3-isopropylmalate dehydrogenase (EC 1.1.1.85)
123	STM0120	STM14_01	CDS	mraW	141119	142060	+	-3.45	-6.48	0.01	0.96	1.05	7.53	S-adenosyl-methyltransferase MraW; similar to Escherichia coli puta	rRNA small subunit methyltransferase H
133	STM0138	STM14_01	CDS	yacG	162976	163167	-	-1.31	-1.99	0.32	3.87	4.49	6.48	zinc-binding protein	FIG003276: zinc-binding protein
134	STM0139	STM14_01	CDS	yacF	163177	163920	-	-1.08	-2.15	0.28	-0.08	-0.23	1.92	hypothetical protein	FIG002842: hypothetical protein
143	STM0149	STM14_01	CDS	uidB [J]_1	172523	173929	-	-0.89	-1.95	0.33	0.43	0.44	2.39	Na+/galactoside symporter	Possible GPH family transporter (TC 2.A.2) for arabinosides
146	STM14_01	STM14_01	sRNA_IV		176795	176951	-	-3.16	-5.65	0.01	0.92	1.98	7.63		
151	STM0159	STM14_01	CDS		189220	190062	-	-1.42	-1.93	0.34	1.38	1.09	3.02	putative restriction endonuclease	HNH endonuclease
153	STM0161	STM14_01	CDS	kdgT	190905	191858	+	-0.87	-2.17	0.28	1.31	2.96	5.13	2-keto-3-deoxygluconate permease; transports degraded pectin prc	2-keto-3-deoxygluconate permease (KDG permease)
162	STM0170	STM14_02	CDS	hpt	201521	202057	+	-1.92	-3.07	0.13	1.42	1.83	4.91	hypoxanthine-guanine phosphoribosyltransferase; Catalyzes the sal	Hypoxanthine-guanine phosphoribosyltransferase (EC 2.4.2.8)
167	STM0175	STM14_02	CDS	stiC	205784	208330	-	-2.29	-3.61	0.08	0.20	0.23	3.84	putativie fimbrial usher; similar to Escherichia coli putative outer m	FIG100795: Fimbriae usher protein StiC
190	STM0198	STM14_02	CDS	stfE	235453	235965	+	-1.46	-3.62	0.08	0.75	1.59	5.21	putative minor fimbrial subunit; minor fimbrial subunit StfE (gi|374	Minor fimbrial subunit StfE
197	STM0206	STM14_02	CDS	btuF	241973	242773	-	-1.35	-4.19	0.05	-0.23	-0.47	3.72	vitamin B12-transporter protein BtuF; solute binding component of	Vitamin B12 ABC transporter, B12-binding component BtuF
198	STM0207	STM14_02	CDS	pfs	242766	243464	-	-1.55	-2.16	0.28	0.42	0.64	2.80	5'-methylthioadenosine/S-adenosylhomocysteine nucleosidase; ena	5'-methylthioadenosine nucleosidase (EC 3.2.2.16) / S-adenosylhomocyst
202	STM0211	STM14_02	CDS	yaeH	248022	248408	-	-0.84	-1.45	0.50	1.76	4.00	5.45	hypothetical protein; similar to Escherichia coli putative structural p	UPF0325 protein YaeH
209	STM0225	STM14_02	CDS	hlpA	265621	266106	+	-5.01	-4.59	0.03	0.08	0.07	4.66	periplasmic chaperone; (SW:OMPH_SALTY); histone-like protein	Outer membrane protein H precursor
215	STM0234	STM14_02	CDS	ldcC	276726	278867	+	-1.11	-3.56	0.08	0.88	2.32	5.89	lysine decarboxylase 2; similar to Escherichia coli lysine decarboxyla	Lysine decarboxylase 2, constitutive (EC 4.1.1.18)
226	STM0246	STM14_02	CDS	yaeE	287077	287730	-	-0.62	-1.85	0.36	0.50	0.55	2.40	DL-methionine transporter permease subunit; part of the MetNIQ n	Methionine ABC transporter permease protein
236	STM0262	STM14_03	CDS	yafS	302852	303574	+	-4.13	-2.60	0.19	-0.30	-0.16	2.43	putative SAM-dependent methyltransferase	FIG005121: SAM-dependent methyltransferase (EC 2.1.1.-)
238	STM0266	STM14_03	CDS	impA [R]	305365	306420	-	-1.08	-3.11	0.12	0.37	0.50	3.60	putative cytoplasmic protein	Uncharacterized protein ImpA
240	STM0268	STM14_03	CDS/sRNA	impG [R]	307423	309306	-	-1.60	-3.95	0.06	1.89	3.04	6.99	putative cytoplasmic protein	Protein ImpG/VasA
242	STM0270	STM14_03	CDS		309813	310637	-	-1.08	-2.52	0.21	-0.13	-0.26	2.26	putative cytoplasmic protein	Protein of avirulence locus ImpE
245	STM0273	STM14_03	CDS	impB [R]	314633	315175	+	-1.78	-2.87	0.15	0.77	1.42	4.29	putative cytoplasmic protein	Uncharacterized protein ImpB
250	STM0277	STM14_03	CDS		318368	318853	+	-2.97	-2.98	0.14	0.32	0.32	3.30	putative cytoplasmic protein	putative cytoplasmic protein
262	STM0290	STM14_03	CDS		332771	333217	+	-2.98	-4.50	0.03	0.06	0.06	4.55	putative cytoplasmic protein	FIG074102: hypothetical protein
292	STM0319	STM14_03	CDS	crl	365213	365614	+	-0.76	-1.58	0.46	0.98	1.82	3.40	DNA-binding transcriptional regulator Crl; involved in the expressior	Curlin genes transcriptional activator
296	STM0324	STM14_03	CDS		369882	370757	-	-0.75	-2.40	0.23	1.92	4.28	6.68	Transposase	Mobile element protein
325	STM0353	STM14_04	CDS	pacS [J]	399253	401541	+	-0.96	-2.38	0.23	0.00	0.00	2.38	putative cation transport ATPase; similar to Escherichia coli putative	Lead, cadmium, zinc and mercury transporting ATPase (EC 3.6.3.3) (EC 3.
357	STM0386	STM14_04	CDS	proC	439949	440758	-	-3.60	-6.93	0.00	0.17	0.16	7.09	pyrroline-5-carboxylate reductase; catalyzes the formation of L-prol	Pyrroline-5-carboxylate reductase (EC 1.5.1.2)
361	STM0390	STM14_04	CDS	aroM	442552	443229	+	-2.72	-4.16	0.05	0.66	0.75	4.92	hypothetical protein; similar to Escherichia coli protein of aro opero	AroM protein
377	STM0406	STM14_04	CDS	yajC	462664	462996	+	-3.33	-2.22	0.27	0.47	0.34	2.56	preprotein translocase subunit YajC; member of preprotein transloc	Preprotein translocase subunit YajC (TC 3.A.5.1.1)
381	STM0410	STM14_04	CDS		466392	467084	+	-1.69	-4.58	0.03	0.61	1.47	6.05	putative regulatory protein	Transcriptional regulator, DeoR family
390	STM0424	STM14_05	CDS	xseB	478109	478351	-	-5.90	-6.87	0.00	-0.02	-0.03	6.84	exodeoxyribonuclease VII small subunit; catalyzes the bidirectional	Exodeoxyribonuclease VII small subunit (EC 3.1.11.6)
391	STM0425	STM14_05	CDS	thiI	478561	480009	+	-2.06	-2.93	0.15	1.21	2.12	5.05	thiamine biosynthesis protein ThiI; Required for the synthesis of the	tRNA S(4)U 4-thiouridine synthase (former ThiI) / Rhodanese-like domain
396	STM0430	STM14_05	CDS	phnR	484042	484761	-	-2.98	-3.40	0.10	0.76	0.79	4.19	2-aminoethylphosphonate transport protein; probable repressor pr	2-aminoethylphosphonate uptake and metabolism regulator
397	STM0431	STM14_05	CDS	phnW	484862	486004	+	-0.73	-1.48	0.49	0.06	0.18	1.66	2-aminoethylphosphonate transport	2-aminoethylphosphonate:pyruvate aminotransferase (EC 2.6.1.37)
452	STM14_05	STM14_05	CDS	ybaN	540406	540783	+	-3.91	-5.79	0.01	0.71	0.92	6.71	hypothetical protein; similar to Escherichia coli putative gene 58 (A	FIG039061: hypothetical protein related to heme utilization
485	STM0515	STM14_06	CDS	allA	576697	577179	+	-1.69	-3.74	0.07	0.72	1.57	5.31	ureidoglycolate hydrolase; catalyzes the formation of glyoxylate fro	Ureidoglycolate hydrolase (EC 3.5.3.19)
498	STM0528	STM14_06	CDS	allD	590674	591723	-	-1.47	-3.25	0.11	-0.03	-0.06	3.19	ureidoglycolate dehydrogenase; similar to Escherichia coli putative	Ureidoglycolate dehydrogenase (EC 1.1.1.154)

517	STM0550	STM14_06	CDS?		612111	612215	+	-0.80	-2.40	0.23	0.35	0.87	3.28	hypothetical protein	
535	STM0569	STM14_06	CDS	ybdG	626329	627576	-	-0.82	-2.18	0.28	0.02	0.03	2.21	hypothetical protein; similar to Escherichia coli putative transport (/	Uncharacterized protein ybdG
560	STM0595	STM14_06	CDS	entC	656949	658124	+	-1.44	-2.63	0.19	-0.16	-0.22	2.41	isochorismate synthase; synthesizes isochorismate acid from chorisı	Isochorismate synthase (EC 5.4.4.2) of siderophore biosynthesis
563	STM0598	STM14_06	CDS	entA	660615	661370	+	-1.27	-2.16	0.28	-0.14	-0.21	1.95	2,3-dihydroxybenzoate-2,3-dehydrogenase; catalyzes the formation	2,3-dihydro-2,3-dihydroxybenzoate dehydrogenase (EC 1.3.1.28) of sider
596	STM0631	STM14_07	CDS	ybeM	694152	694940	+	-0.93	-1.73	0.41	0.40	0.66	2.39	putative hydrolase; similar to Escherichia coli putative amidase (AA(	Aliphatic amidase AmiE (EC 3.5.1.4)
607	STM0644	STM14_07	CDS	cobD_1	706273	707367	+	-0.94	-1.84	0.37	0.51	1.12	2.96	threonine-phosphate decarboxylase; cobalamin biosynthesis protei	L-threonine 3-O-phosphate decarboxylase (EC 4.1.1.81)
630	STM0669	STM14_07	CDS	phoL	733733	734818	-	-1.32	-2.52	0.21	-0.03	-0.02	2.49	putative phosphate starvation-inducible protein; similar to Escheric	Phosphate starvation-inducible protein PhoH, predicted ATPase
637	STM0683	STM14_07	CDS		743320	743448	+	-5.99	-8.28	0.00	0.43	0.36	8.64	hypothetical protein	
655	STM0703	STM14_08	CDS	kdpD	767510	770194	-	-1.46	-1.67	0.43	1.34	1.64	3.31	sensor protein KdpD; sensory histidine kinase in two-component re	Osmosensitive K+ channel histidine kinase KdpD (EC 2.7.3.-)
670	STM0719	STM14_08	CDS	glf	784487	785665	+	-2.23	-3.39	0.10	0.91	0.88	4.27	UDP-galactopyranose mutase	UDP-galactopyranose mutase (EC 5.4.99.9)
675	STM0724	STM14_08	CDS		789411	791189	+	-1.93	-2.77	0.17	0.49	0.58	3.35	putative glycosyltransferase; cell wall biogenesis	putative glycosyltransferase
687	STM0737	STM14_08	CDS	sucB	805058	806266	+	-1.56	-2.03	0.31	-0.12	-0.25	1.78	dihydrolipoamide acetyltransferase; component of 2-oxoglutarate c	Dihydrolipoamide succinyltransferase component (E2) of 2-oxoglutarate
716	STM0772	STM14_08	CDS	gpmA	836703	837455	-	-1.45	-1.90	0.35	0.06	0.09	1.99	phosphoglyceromutase; catalyzes the interconversion of 2-phospho	Phosphoglycerate mutase (EC 5.4.2.1)
717	STM0773	STM14_08	CDS	galM	837679	838719	-	-1.48	-3.77	0.07	0.04	0.06	3.84	aldose 1-epimerase; catalyzes the conversion of alpha-aldose to the	Aldose 1-epimerase (EC 5.1.3.3)
719	STM0775	STM14_08	CDS	galT	839864	840910	-	-2.87	-3.58	0.08	0.11	0.11	3.69	galactose-1-phosphate uridylyltransferase; catalyzes the interconve	Galactose-1-phosphate uridylyltransferase (EC 2.7.7.10)
727	STM0783	STM14_09	CDS	modC	847480	848538	+	-0.95	-2.08	0.30	0.55	1.01	3.09	molybdate transporter ATP-binding protein; Part of the ABC transpc	Molybdenum transport ATP-binding protein ModC (TC 3.A.1.8.1)
728	STM0784	STM14_09	CDS	ybhA	848539	849399	-	-0.77	-1.72	0.41	0.88	1.73	3.45	Putative phosphatase	Putative phosphatase
729	STM0785	STM14_09	CDS	ybhE	849521	850516	+	-1.14	-2.67	0.18	-0.25	-0.27	2.40	6-phosphogluconolactonase; catalyzes the hydrolysis of 6-phosphog	6-phosphogluconolactonase (EC 3.1.1.31)
745	STM0800	STM14_09	CDS	slrP	867285	869582	+	-0.75	-1.56	0.46	0.66	0.61	2.17	leucine-rich repeat-containing protein; leucine-rich repeat protein S	invasion plasmid antigen / internalin, putative
780	rybA	STM14_09	sRNA_JV	RybA [P]	903092	903180	-	-1.60	-2.67	0.18	0.52	0.70	3.37		
783	STM0837	STM14_09	CDS	erfK [J]	904949	905869	-	-1.41	-3.29	0.11	-0.04	-0.04	3.25	hypothetical protein	L,D-transpeptidase YbiS
808	STM0862	STM14_10	CDS	yliJ	935679	936305	-	-0.84	-2.29	0.25	-0.14	-0.30	2.00	putative glutathione S-transferase; similar to Escherichia coli putati	Uncharacterized glutathione S-transferase-like protein
841	STM0931	STM14_10	CDS	ybjR	964016	964846	+	-0.98	-1.96	0.33	-0.07	-0.11	1.85	putative aminidase; similar to Escherichia coli putative regulator (A/	N-acetylmuramoyl-L-alanine amidase (EC 3.5.1.28)
853	STM14_10	STM14_10	CDS	cspD	980013	980321	-	-1.67	-2.65	0.18	0.20	0.30	2.95	hypothetical protein; cold-shock domain family protein-related prot	
918	STM2613	STM14_11	CDS		1073250	1073585	+	-2.02	-3.10	0.13	0.64	0.90	4.00	hypothetical protein	Phage holin
928	STM1037	STM14_11	CDS		1082356	1082757	+	-2.36	-4.22	0.05	0.19	0.24	4.45	minor tail protein	Phage minor tail protein
929	STM1038	STM14_11	CDS		1082769	1083518	+	-1.14	-2.78	0.17	0.49	0.99	3.77	major tail protein	Phage tail assembly
963	STM1072	STM14_12	CDS	tfoX [R]	1121444	1122049	+	-1.41	-1.90	0.35	-0.14	-0.36	1.55	putative DNA transformation protein	DNA transformation protein TfoX
996	STM1106	STM14_12	CDS	hpaI	1151517	1152308	+	-2.29	-3.24	0.11	0.82	1.12	4.36	4-hydroxyphenylacetate catabolism	2,4-dihydroxyhept-2-ene-1,7-dioic acid aldolase (EC 4.1.2.-)
1033	STM1143	STM14_13	CDS	csgB	1189295	1189750	+	-1.87	-1.95	0.33	-0.40	-0.29	1.66	curlin minor subunit; CsgB; functions as a nucleator in the assembly	Minor curlin subunit CsgB, nucleation component of curlin monomers
1041	STM1151	STM14_13	CDS	mdoH	1196078	1198651	+	-4.75	-6.50	0.01	0.00	0.00	6.50	periplasmic glucans biosynthesis protein MdoH	Glucans biosynthesis glucosyltransferase H (EC 2.4.1.-)
1053	STM1163	STM14_13	CDS	pyrC	1206677	1207723	-	-2.82	-3.22	0.11	-0.23	-0.25	2.97	dihydroorotase; catalyzes the formation of N-carbamoyl-L-aspartate	Dihydroorotase (EC 3.5.2.3)
1070	STM1180	STM14_13	CDS	flgH	1220800	1221507	+	-1.11	-2.24	0.26	1.04	1.80	4.04	flagellar L-ring protein FlgH	Flagellar L-ring protein FlgH
1082	STM1191	STM14_13	CDS	rpmF	1234009	1234182	+	-2.49	-4.58	0.03	0.00	0.00	4.58	50S ribosomal protein L32; some L32 proteins have zinc finger motil	LSU ribosomal protein L32p
1085	STM1197	STM14_13	CDS	fabF	1238598	1239839	+	-2.55	-3.87	0.06	0.02	0.03	3.90	3-oxoacyl-(acyl carrier protein) synthase II; FabF; beta-ketoacyl-ACP	3-oxoacyl-[acyl-carrier-protein] synthase, KASII (EC 2.3.1.41)
1100	STM1214	STM14_13	CDS	ycfR	1255525	1255848	+	-1.13	-2.72	0.17	0.91	0.98	3.70	putative outer membrane protein	Putative outer membrane protein
1118	STM1233	STM14_14	CDS	ycfC	1279735	1280382	-	-2.10	-5.48	0.01	0.10	0.23	5.71	hypothetical protein; HflD; UPF0274; in Escherichia coli this protein	FIG002903: a protein of unknown function perhaps involved in purine me
1172	STM1274	STM14_15	CDS	yeaQ	1363114	1363362	+	-1.25	-1.62	0.44	0.91	0.71	2.33	hypothetical protein	Transglycosylase associated protein
1256	STM1360	STM14_16	CDS		1451235	1451360	-	-1.48	-2.66	0.18	0.36	0.94	3.60	hypothetical protein	
1327	STM1431	STM14_17	CDS		1519171	1519452	+	-1.91	-3.59	0.08	-0.30	-0.27	3.31	hypothetical protein	hypothetical protein
1359	STM14_17	STM14_17	pseudo	malX	1548839	1549058	-	-1.12	-1.91	0.35	1.29	2.71	4.62	pseudogene; frameshift	
1384	STM1490	STM14_18	CDS	bioD_2	1576425	1577120	+	-1.12	-1.46	0.50	1.61	3.48	4.94	putative dithiobiotin synthetase; DTB synthetase; dethiobiotin syntl	Dethiobiotin synthetase (EC 6.3.3.3)
1429	STM1537	STM14_18	CDS		1621279	1622022	-	-1.33	-2.39	0.23	-0.08	-0.16	2.22	putative Ni/Fe hydrogenase 1 b-type cytochrome subunit; similar to	Ni,Fe-hydrogenase I cytochrome b subunit
1432	STM1540	STM14_18	CDS		1625310	1626401	+	-2.38	-4.17	0.05	0.39	0.43	4.60	putative hydrolase	Choloylglycine hydrolase (EC 3.5.1.24)
1450	STM1557	STM14_18	CDS		1644722	1645924	+	-1.61	-2.74	0.17	-0.32	-0.29	2.45	putative aminotransferase; similar to Escherichia coli enzyme that n	Aspartate aminotransferase (EC 2.6.1.1)
1466	STM1573	STM14_18	CDS		1666322	1666762	+	-1.91	-2.80	0.16	0.53	0.47	3.27	putative cytoplasmic protein	Gfa-like protein
1492	STM1599	STM14_19	CDS	pdgL	1699931	1700701	-	-1.64	-3.61	0.08	0.44	0.82	4.43	periplasmic dipeptidase precursor; D-alanyl-D-alanine dipeptidase f	D-alanyl-D-alanine dipeptidase (EC 3.4.13.22)
1605	STM14_20	STM14_20	CDS	pyrF	1811276	1812073	-	-2.69	-2.41	0.23	0.27	0.27	2.68	Orotidine 5'-phosphate decarboxylase (EC 4.1.1.23); De Novo I	Orotidine 5'-phosphate decarboxylase (EC 4.1.1.23)
1631	STM1735	STM14_20	CDS	ispZ [J]	1840788	1841327	+	-0.95	-2.09	0.30	0.12	0.08	2.17	intracellular septation protein A; Involved in cell division; probably i	Intracellular septation protein IspA
1651	STM1755	STM14_21	CDS	ychJ	1861748	1862206	+	-1.72	-1.92	0.34	0.80	0.58	2.50	hypothetical protein	UPF0225 protein YchJ
1668	STM1774	STM14_21	CDS	sirC	1882801	1883190	-	-1.95	-2.73	0.17	0.63	0.51	3.24	putative transcriptional regulator; regulation of invasion genes; (gi |	FIG002082: Protein SirB2
1691	STM1799	STM14_21	CDS	emtA	1908705	1909316	-	-1.40	-3.56	0.08	-0.08	-0.18	3.38	membrane-bound lytic murein transglycosylase E; similar to Escheri	Membrane-bound lytic murein transglycosylase E (EC 3.2.1.-)
1692	STM1800	STM14_21	CDS	ldcA	1909487	1910401	+	-4.12	-7.31	0.00	0.04	0.04	7.35	L,D-carboxypeptidase A; catalyzes the release of D-alanine from L-al	Muramoyltetrapeptide carboxypeptidase (EC 3.4.17.13)
1706	STM1814	STM14_21	CDS	minC	1922776	1923483	+	-2.63	-5.29	0.02	0.20	0.22	5.50	septum formation inhibitor; blocks the formation of polar Z-ring sep	Septum site-determining protein MinC
1729	STM1836	STM14_22	CDS	ftsI [D]	1944987	1946732	-	-0.83	-1.47	0.50	0.45	0.71	2.18	putative penicillin-binding protein 3; similar to Escherichia coli septu	Cell division protein FtsI [Peptidoglycan synthetase] (EC 2.4.1.129)
1732	STM1839	STM14_22	CDS		1947966	1948253	-	-2.93	-2.58	0.19	0.54	1.09	3.68	hypothetical protein; putative periplasmic protein	FIG00638740: hypothetical protein
1767	STM1870	STM14_22	CDS		1977760	1978413	+	-2.50	-2.01	0.31	-0.25	-0.37	1.65	RecE-like protein; exoVIII; similar to Escherichia coli exonuclease VII	FIG01047716: hypothetical protein
1797	STM1898	STM14_23	CDS	ruvC	2004632	2005153	-	-3.51	-6.99	0.00	0.38	0.82	7.81	Holliday junction resolvase; endonuclease; resolves Holliday structu	Crossover junction endodeoxyribonuclease RuvC (EC 3.1.22.4)
1801	STM1902	STM14_23	CDS	entB [J]	2008612	2009178	+	-1.62	-2.90	0.15	0.24	0.51	3.41	hypothetical protein	Nicotinamidase/isochorismatase family protein
1815	STM1916	STM14_23	CDS	cheY	2023687	2024076	-	-0.76	-1.44	0.50	0.01	0.01	1.46	chemotaxis regulatory protein CheY; chemotaxis regulator that, wh	Chemotaxis regulator - transmits chemoreceptor signals to flagelllar mot
1816	STM1917	STM14_23	CDS	cheB	2024094	2025143	-	-1.41	-2.38	0.23	1.62	2.66	5.03	chemotaxis-specific methylesterase; regulates chemotaxis by deme	Chemotaxis response regulator protein-glutamate methylesterase CheB (
1821	STM1922	STM14_23	CDS	motB	2030602	2031531	-	-3.77	-4.77	0.03	0.17	0.20	4.97	flagellar motor protein MotB; with MotA forms the ion channels tha	Flagellar motor rotation protein MotB
1823	STM1924	STM14_23	CDS	flhC	2032540	2033118	-	-0.80	-2.59	0.19	-0.48	-0.47	2.12	transcriptional activator FlhC; With FlhD is involved in the activation	Flagellar transcriptional activator FlhC
1843	STM1948	STM14_23	CDS		2050620	2050775	+	-3.59	-2.98	0.14	1.31	1.86	4.84	putative inner membrane protein	FIG01045369: hypothetical protein
1864	STM1970	STM14_23	CDS	fliG	2069619	2070614	+	-2.41	-4.34	0.04	1.86	3.11	7.45	flagellar motor switch protein G; One of three proteins involved in s	Flagellar motor switch protein FliG
1869	STM1975	STM14_23	CDS	fliL	2074408	2074935	+	-2.12	-3.42	0.09	0.44	0.79	4.21	flagellar flil protein. {salmonella	Flagellar biosynthesis protein FliL

1886	STM1992	STM14_24	CDS	vsr	2085283	2085753	-	-0.99	-2.52	0.21	0.66	0.67	3.19	DNA mismatch endonuclease; similar to Escherichia coli DNA misma	Very-short-patch mismatch repair endonuclease (G-T specific)
1892	STM1996	STM14_24	CDS	cspB	2090630	2090842	-	-2.44	-3.15	0.12	-0.61	-0.53	2.63	putative cold-shock protein; similar to Escherichia coli cold shock-lik	Cold shock protein CspG
1897	STM0906	STM14_24	CDS		2117454	2117798	-	-1.76	-2.44	0.22	0.27	0.54	2.98	lysis protein (holin); similar to Xenorhabdus nematophila ; CAB5844	Phage holin
1899	STM2003	STM14_24	pseudo	intL [J]_1	2135043	2135361	+	-3.85	-3.97	0.06	0.65	0.48	4.45	pseudogene; frameshift	
1905	STM2009	STM14_24	CDS	amn	2142858	2144312	-	-0.74	-1.66	0.43	0.17	0.18	1.84	AMP nucleosidase; Catalyzes the hydrolysis of AMP to form adenine	AMP nucleosidase (EC 3.2.2.4)
1906	STM2010	STM14_24	CDS		2144358	2144519	+	-1.78	-2.25	0.26	0.23	0.18	2.43	putative cytoplasmic protein	
1907	STM2011	STM14_24	CDS		2144508	2144834	-	-1.83	-3.49	0.09	0.23	0.18	3.67	putative cytoplasmic protein; similar to STM2508 and STM2902	Putative acyl carrier protein
1916	STM2022	STM14_25	CDS	cbiQ	2153574	2154254	-	-0.99	-1.71	0.41	-0.11	-0.25	1.46	membrane protein	Transmembrane component CbiQ of energizing module of cobalt ECF tra
1937	STM2043	STM14_25	CDS	pduG	2172405	2174237	+	-0.82	-1.49	0.49	0.19	0.32	1.81	propanediol dehydratase reactivation protein; propanediol utilizatio	Propanediol dehydratase reactivation factor large subunit
1939	STM2045	STM14_25	CDS	pduJ	2174596	2174871	+	-0.79	-2.06	0.31	0.12	0.31	2.37	polyhedral body protein; similar to Escherichia coli detox protein (A	Propanediol utilization polyhedral body protein PduJ
1975	STM2081	STM14_25	CDS	gnd	2210913	2212319	-	-1.73	-3.52	0.09	0.29	0.35	3.87	6-phosphogluconate dehydrogenase; catalyzes the formation of D-r	6-phosphogluconate dehydrogenase, decarboxylating (EC 1.1.1.44)
1997	STM2103	STM14_25	CDS	wcaJ	2237124	2238518	-	-1.12	-2.85	0.16	0.36	0.80	3.64	putative UDP-glucose lipid carrier transferase; WcaJ; glucose-1-pho	Colanic acid biosynthsis UDP-glucose lipid carrier transferase WcaJ
1999	STM2105	STM14_25	CDS		2239941	2240216	+	-1.36	-2.17	0.28	1.11	1.94	4.11	hypothetical protein	hypothetical protein
2002	STM2108	STM14_26	CDS	wcaG	2243188	2244153	-	-1.27	-2.34	0.24	-0.01	-0.01	2.32	GDP-fucose synthetase; similar to Escherichia coli putative nucleotic	GDP-L-fucose synthetase (EC 1.1.1.271)
2039	STM2142	STM14_26	CDS	yegT	2287779	2289095	+	-0.67	-1.68	0.42	0.42	0.85	2.54	Putative nucleoside transporter yegT	Putative nucleoside transporter YegT
2056	STM2159	STM14_26	CDS	lytS	2306013	2307800	-	-1.01	-2.18	0.28	-0.26	-0.54	1.64	putative sensor kinase; similar to Escherichia coli putative 2-compor	Autolysis histidine kinase LytS
2069	STM2172	STM14_26	CDS	mdtQ [D]	2320382	2321818	-	-1.98	-3.81	0.07	-0.15	-0.22	3.59	multidrug resistance outer membrane protein MdtQ; may be involv	RND efflux system, outer membrane lipoprotein, NodT family
2098	STM2201	STM14_27	CDS	yeiE	2352100	2352963	-	-2.07	-2.26	0.26	-0.15	-0.18	2.08	putative DNA-binding transcriptional regulator; similar to Escherichi	LysR family transcriptional regulator YeiE
2115	STM2217	STM14_27	CDS	oppB_2	2369587	2370681	+	-1.11	-2.38	0.23	1.52	3.16	5.54	putative ABC-type dipeptide/oligopeptide/nickel transport system p	Oligopeptide transport system permease protein OppB (TC 3.A.1.5.1)
2131	STM2234	STM14_27	CDS		2386387	2386914	-	-2.42	-3.51	0.09	0.00	0.00	3.51	putative phage tail fiber assembly protein	FIG01047148: hypothetical protein
2147	STM2257	STM14_27	CDS	napH	2406413	2407282	-	-0.89	-1.46	0.50	0.62	1.24	2.70	quinol dehydrogenase membrane component; part of NapHG quinc	Polyferredoxin NapH (periplasmic nitrate reductase)
2150	STM2260	STM14_27	CDS	napD	2410454	2410717	-	-2.29	-4.42	0.04	0.48	0.56	4.97	assembly protein for periplasmic nitrate reductase	periplasmic diheme c-type cytochrome, NapB
2173	STM2284	STM14_28	CDS	glpA	2441789	2443417	+	-1.67	-2.66	0.18	-0.31	-0.41	2.25	sn-glycerol-3-phosphate dehydrogenase subunit A; anaerobic, catal	Anaerobic glycerol-3-phosphate dehydrogenase subunit A (EC 1.1.5.3)
2180	STM2290	STM14_28	CDS	yfaV	2448422	2449711	-	-2.12	-2.67	0.18	0.04	0.04	2.71	putative transport protein; similar to Escherichia coli putative trans	L-rhamnonate transporter (predicted by genome context)
2197	STM2307	STM14_28	CDS	menB	2465552	2466409	-	-2.28	-3.95	0.06	1.14	1.82	5.77	naphthoate synthase; catalyzes the formation of 1,4-dihydroxy-2-na	Naphthoate synthase (EC 4.1.3.36)
2235	STM2345	STM14_28	CDS	purR [D]	2508935	2509954	+	-1.47	-2.03	0.31	0.02	0.03	2.06	putative transcriptional regulator; similar to Escherichia coli regulat	Transcriptional regulator, LacI family
2244	STM2354	STM14_29	CDS	hisJ	2516043	2516825	-	-1.46	-4.38	0.04	-0.35	-0.52	3.86	histidine transport protein; histidine-binding periplasmic protein pr	Histidine ABC transporter, histidine-binding periplasmic protein precurso
2266	STM2377	STM14_29	CDS		2539420	2540076	-	-1.05	-1.64	0.44	-0.18	-0.15	1.49	putative inner membrane protein	Putative inner membrane protein
2312	STM2431	STM14_29	CDS	ptsH	2596138	2596395	+	-3.73	-3.03	0.13	0.60	0.75	3.78	phosphohistidinoprotein-hexose phosphotransferase component o	Phosphotransferase system, phosphocarrier protein HPr
2316	STM2436	STM14_29	CDS	ptsJ	2600055	2601347	+	-1.71	-3.21	0.11	0.46	0.68	3.88	putative regulatory protein; putative transcriptional regulator PtsJ (	Predicted transcriptional regulator of pyridoxine metabolism
2321	STM2441	STM14_29	CDS	cysA	2604182	2605279	-	-1.05	-2.52	0.21	0.02	0.03	2.55	sulfate/thiosulfate transporter subunit; sulfate transport atp-bindin	Sulfate and thiosulfate import ATP-binding protein CysA (EC 3.6.3.25)
2330	STM2450	STM14_30	CDS	amiA	2611885	2612754	+	-1.55	-2.70	0.18	0.53	1.54	4.24	N-acetylmuramoyl-l-alanine amidase I; probable N-acetylmuramoyl	N-acetylmuramoyl-L-alanine amidase (EC 3.5.1.28)
2337	STM2457	STM14_30	CDS	eutC	2617888	2618784	-	-0.97	-2.37	0.23	-0.23	-0.55	1.82	ethanolamine ammonia-lyase small subunit; catalyzes the formatio	Ethanolamine ammonia-lyase light chain (EC 4.3.1.7)
2338	STM2458	STM14_30	CDS	eutB	2618803	2620164	-	-1.51	-2.43	0.22	-0.17	-0.22	2.21	ethanolamine ammonia-lyase heavy chain; ethanolamine ammonia-	Ethanolamine ammonia-lyase heavy chain (EC 4.3.1.7)
2358	STM2479	STM14_30	CDS	aegA	2641167	2643128	-	-0.83	-2.63	0.19	-0.07	-0.07	2.57	putative oxidoreductase Fe-S binding subunit; unknown function; in	Glutamate synthase [NADPH] small chain (EC 1.4.1.13)
2365	STM2486	STM14_30	CDS	ypfJ [R]	2652662	2653525	-	-1.62	-2.87	0.15	-0.01	-0.01	2.86	putative inner membrane protein	YpfJ protein, zinc metalloprotease superfamily
2373	STM2495	STM14_30	CDS	arsC [J]	2661026	2661385	+	-1.77	-3.14	0.12	0.57	1.22	4.35	putative arsenate reductase; similar to Escherichia coli putative oxic	Arsenate reductase (EC 1.20.4.1)
2379	STM2501	STM14_30	CDS	ppk	2666489	2668555	+	-1.00	-1.87	0.36	-0.21	-0.34	1.53	polyphosphate kinase; catalyzes the reversible transfer of the termi	Polyphosphate kinase (EC 2.7.4.1)
2383	STM2505	STM14_30	CDS		2672655	2672915	-	-1.40	-2.73	0.17	0.75	0.95	3.68	putative inner membrane protein	FIG01047445: hypothetical protein
2398	STM2520	STM14_30	CDS	yfgL	2704600	2705778	-	-2.08	-4.18	0.05	0.35	0.75	4.93	outer membrane protein assembly complex subunit YfgL; with YaeT	Outer membrane protein YfgL, lipoprotein component of the protein ass
2402	STM2524	STM14_30	CDS	yfgA	2708953	2709957	-	-3.58	-9.02	0.00	0.25	0.42	9.44	hypothetical protein; similar to Escherichia coli putative membrane	FIG021952: putative membrane protein
2407	STM2529	STM14_31	CDS	dmsB [J]_1	2713839	2714468	-	-1.70	-3.20	0.11	-0.10	-0.21	2.99	putative anaerobic dimethylsulfoxide reductase; similar to Escherich	Anaerobic dimethyl sulfoxide reductase chain B (EC 1.8.5.3)
2420	STM2541	STM14_31	CDS	iscA	2732323	2732646	-	-2.83	-2.75	0.17	0.40	0.55	3.30	iron-sulfur cluster assembly protein; forms iron-sulfur clusters of fe	Iron binding protein IscA for iron-sulfur cluster assembly
2422	STM2544	STM14_31	CDS	iscR	2734484	2734978	-	-1.49	-2.34	0.24	0.56	0.58	2.93	DNA-binding transcriptional regulator IscR; regulates the expression	Iron-sulfur cluster regulator IscR
2426	STM2549	STM14_31	CDS	asrB	2739043	2739861	+	-2.25	-2.94	0.15	-0.18	-0.15	2.79	anaerobic sulfite reductase subunit B; with AsrAC catalyzes the redu	Anaerobic sulfite reductase subunit B
2458	STM2583	STM14_31	CDS	lepA	2778325	2780124	-	-3.33	-6.34	0.01	-0.64	-0.53	5.81	GTP-binding protein LepA; binds to the ribosome on the universally	Translation elongation factor LepA
2488	STM2660	STM14_32	CDS	clpB_2	2856033	2858606	-	-5.00	-5.08	0.02	0.63	0.68	5.76	protein disaggregation chaperone; similar to Escherichia coli heat sh	ClpB protein
2490	STM2662	STM14_32	CDS	rluD	2859464	2860444	-	-5.37	-6.91	0.00	2.15	1.32	8.24	23S rRNA pseudouridine synthase D; responsible for synthesis of ps	Ribosomal large subunit pseudouridine synthase D (EC 4.2.1.70)
2492	STM2664	STM14_32	pseudo		2861349	2861548	+	-1.05	-2.18	0.28	0.38	0.40	2.58	pseudogene; two in-frame stops relative to Escherichia coli b2596	
2532	STM2752	STM14_33	CDS		2910335	2911315	+	-1.64	-4.36	0.04	0.37	0.86	5.22	putative glucitol-specific PTS enzyme III; similar to Escherichia coli P	PTS system, glucitol/sorbitol-specific IIB component and second of two II
2539	STM2759	STM14_33	CDS		2917868	2919547	+	-1.21	-2.69	0.18	0.58	0.61	3.30	putative dipeptide/oligopeptide/nickel ABC-type transport system p	putative dipeptide/oligopeptide/nickel ABC-type transport system peripl
2571	STM2791	STM14_33	CDS	gabD	2960369	2961817	+	-0.75	-1.70	0.42	0.27	0.50	2.20	succinate-semialdehyde dehydrogenase I; catalyzes the formation o	Succinate-semialdehyde dehydrogenase [NAD(P)+] (EC 1.2.1.16)
2573	STM2793	STM14_33	CDS	gabP	2963245	2964645	+	-1.59	-3.29	0.11	0.75	1.41	4.69	gamma-aminobutyrate transporter; similar to Escherichia coli trans	gamma-aminobutyrate (GABA) permease
2579	STM2799	STM14_33	CDS	stpA	2967460	2967861	-	-2.90	-6.39	0.01	0.40	0.62	7.01	DNA binding protein, nucleoid-associated; DNA-binding protein STP	DNA-binding protein H-NS
2588	STM2807	STM14_33	CDS	nrdE	2972717	2974861	+	-1.47	-1.56	0.46	0.35	0.78	2.33	ribonucleotide-diphosphate reductase subunit alpha; Catalyzes the	Ribonucleotide reductase of class Ib (aerobic), alpha subunit (EC 1.17.4.1
2607	STM2832	STM14_34	CDS	srlA	2998543	2999106	+	-0.71	-1.83	0.37	0.22	0.44	2.27	glucitol/sorbitol-specific enzyme IIC component; similar to Escherich	PTS system, glucitol/sorbitol-specific IIC component (EC 2.7.1.69)
2620	STM2844	STM14_34	CDS		3011104	3011913	-	-1.72	-2.87	0.15	0.87	1.59	4.46	hypothetical protein; putative periplasmic protein	Putative periplasmic or exported protein
2633	STM2857	STM14_34	CDS	hypD	3021593	3022714	+	-1.46	-2.67	0.18	0.57	1.12	3.79	putative hydrogenase formation protein; similar to Escherichia coli	[NiFe] hydrogenase metallocenter assembly protein HypD
2638	STM2862	STM14_34	CDS	sitB	3027520	3028341	+	-1.19	-2.06	0.31	0.38	0.88	2.94	putative ATP-binding protein; SitB (gi|5231095); Salmonella iron tra	Manganese ABC transporter, ATP-binding protein SitB
2687	STM2909	STM14_35	CDS	mutS	3070516	3073083	+	-2.06	-3.76	0.07	0.49	0.84	4.60	DNA mismatch repair protein; This protein performs the mismatch r	DNA mismatch repair protein MutS
2701	STM2924	STM14_35	CDS	rpoS	3085731	3086723	-	-2.23	-4.69	0.03	0.63	0.53	5.22	RNA polymerase sigma factor RpoS; sigma factors are initiation fact	RNA polymerase sigma factor RpoS
2704	STM2927	STM14_35	CDS	surE	3088715	3089476	-	-3.09	-3.34	0.10	1.31	1.31	4.65	stationary phase survival protein SurE; catalyzes the conversion of a	5-nucleotidase SurE (EC 3.1.3.5) @ Exopolyphosphatase (EC 3.6.1.11)
2705	STM2928	STM14_35	CDS	surE	3088715	3089476	-	-4.00	-3.26	0.11	0.45	0.24	3.51	stationary phase survival protein SurE; catalyzes the conversion of a	5-nucleotidase SurE (EC 3.1.3.5) @ Exopolyphosphatase (EC 3.6.1.11)
2748	STM2972	STM14_35	CDS	xni	3143407	3144222	+	-0.94	-1.79	0.39	1.05	2.21	4.00	exonuclease IX; has 3'-5' exonuclease activity that preferentially act	DNA polymerase I (EC 2.7.7.7)
2767	STM2992	STM14_36	CDS	argA	3162651	3163982	+	-3.22	-5.98	0.01	0.94	1.67	7.65	N-acetylglutamate synthase; catalyzes the formation of N-acetyl-L-g	N-acetylglutamate synthase (EC 2.3.1.1)
2771	STM2996	STM14_36	CDS	recC	3172521	3175892	-	-1.83	-4.24	0.05	-0.02	-0.05	4.19	exonuclease V subunit gamma; catalyzes ATP-dependent exonucleo	Exodeoxyribonuclease V gamma chain (EC 3.1.11.5)

2781	STM3006	STM14_36	CDS	terC [R]_1	3184067	3184780	+	-1.75	-2.73	0.17	-0.47	-0.42	2.31	putative transport protein; similar to Escherichia coli putative transp	FIG003462: membrane protein
2782	STM3007	STM14_36	CDS		3184932	3185273	-	-0.85	-2.18	0.28	0.24	0.46	2.65	hypothetical protein	
2807	STM3028	STM14_36	CDS	stdB	3208362	3210851	-	-1.56	-3.33	0.10	-0.11	-0.26	3.07	putative outer membrane usher protein; similar to Escherichia coli p	FIG036507: Fimbriae usher protein StdB
2812	STM3034	STM14_36	CDS	trbH [R]	3214631	3214963	+	-2.09	-4.62	0.03	0.55	0.77	5.39	IncF plasmid conjugative transfer protein TrbH	
2851	STM3073	STM14_37	CDS		3253466	3254173	+	-2.20	-3.05	0.13	-0.09	-0.16	2.89	putative ABC-type cobalt transport system permease component; C	Transmembrane component STY3231 of energizing module of queuosine
2857	STM3079	STM14_37	CDS	menX [R]	3259783	3260598	-	-2.59	-3.74	0.07	0.18	0.21	3.95	putative hydrolase/acyltransferase	FIG01048344: hypothetical protein
2862	STM3084.S	STM14_37	CDS		3266219	3266968	-	-1.98	-3.66	0.08	0.31	0.61	4.27	putative regulatory protein; similar to Escherichia coli regulator for	Hexuronate utilization operon transcriptional repressor ExuR
2863	STM3085	STM14_37	CDS		3267030	3267236	-	-1.89	-2.30	0.25	0.82	2.10	4.40	hypothetical protein	
2866	STM14_37	STM14_37	?	yqgC	3270285	3270503	+	-1.11	-3.15	0.12	0.74	1.86	5.02		
2867	STM3088	STM14_37	?	yqgC	3270285	3270503	+	-1.14	-3.68	0.07	0.74	2.14	5.82		
2870	STM3092	STM14_37	CDS	sprT	3273934	3274431	+	-1.65	-3.45	0.09	-0.21	-0.23	3.22	hypothetical protein	Protein sprT
2881	STM3103	STM14_37	CDS	yggV	3281650	3282243	+	-1.95	-4.21	0.05	1.08	1.52	5.74	putative deoxyribonucleotide triphosphate pyrophosphatase; HAM1	Nucleoside 5-triphosphatase RdgB (dHAPTP, dITP, XTP-specific) (EC 3.6.1.
2912	STM3136	STM14_37	CDS	mtlK [J]_3	3315674	3317146	+	-1.57	-2.93	0.15	0.15	0.31	3.24	putative D-mannonate oxidoreductase; similar to Escherichia coli D-	D-mannonate oxidoreductase (EC 1.1.1.57)
2915	STM3139	STM14_38	CDS	gsp	3320598	3322454	-	-2.06	-4.07	0.05	0.54	0.79	4.86	bifunctional glutathionylspermidine amidase/glutathionylspermidin	Glutathionylspermidine synthase (EC 6.3.1.8) / Glutathionylspermidine ar
2925	STM3149	STM14_38	CDS	hybA	3329906	3330892	-	-1.10	-3.10	0.13	0.70	2.17	5.26	hydrogenase 2 protein HybA; Fe-S ferrodoxin type component; part	Fe-S-cluster-containing hydrogenase components 1
2947	STM3171	STM14_38	CDS	ygiK	3351367	3352674	+	-1.60	-2.05	0.31	1.06	1.44	3.49	putative transporter; hypothetical 46.1 kDa protein in plsC 3'region	TRAP-type C4-dicarboxylate transport system, large permease componer
2953	STM3178	STM14_38	CDS	qseC	3359706	3361055	+	-3.54	-5.58	0.01	0.49	0.54	6.12	sensor protein QseC; similar to Escherichia coli putative 2-compone	Sensory histidine kinase QseC
2956	STM3182	STM14_38	CDS	yqiA	3364134	3364715	-	-1.15	-3.11	0.12	0.58	0.74	3.85	esterase YqiA; Displays esterase activity toward palmitoyl-CoA and p	Putative esterase, FIGfam005057
2957	STM3183	STM14_38	CDS	icc	3364715	3365542	-	-1.84	-2.76	0.17	0.91	1.53	4.29	cyclic 3',5'-adenosine monophosphate phosphodiesterase; similar to	3',5'-cyclic-nucleotide phosphodiesterase (EC 3.1.4.17)
2971	STM3198	STM14_38	CDS		3378034	3378654	+	-1.49	-3.02	0.13	0.91	2.01	5.03	putative inner membrane protein; similar to Escherichia coli putativ	Putative inner membrane protein
2974	rygD	STM14_38	sRNA_JV	RygD [P]	3380580	3380727	-	-1.25	-2.27	0.26	1.60	2.97	5.24		
2994	STM3222	STM14_39	CDS	sanA_2	3408478	3409170	+	-1.32	-3.05	0.13	2.02	3.88	6.93	putative integral membrane protein	SanA protein
3018	STM3245	STM14_39	CDS	tdcA	3430726	3431664	-	-1.02	-1.67	0.43	-0.05	-0.05	1.62	DNA-binding transcriptional activator TdcA; regulates the tdcABCDE	Threonine catabolic operon transcriptional activator TdcA
3025	STM3252	STM14_39	CDS	agaR	3439691	3440524	-	-2.65	-2.42	0.22	-0.42	-0.45	1.97	AGA operon transcriptional repressor; similar to Escherichia coli put	Transcriptional repressor of aga operon
3032	STM3264	STM14_39	CDS	yraM	3446511	3448553	+	-0.91	-1.69	0.42	1.09	1.55	3.24	putative transglycosylase; similar to Escherichia coli putative glycosy	LppC putative lipoprotein
3041	STM3273	STM14_39	CDS	yhbT	3452716	3453240	-	-1.43	-2.74	0.17	1.42	1.56	4.29	putative lipid carrier protein	FIG138517: Putative lipid carrier protein
3049	STM3281	STM14_39	CDS	nlpI	3461437	3462321	-	-2.19	-2.72	0.17	-0.23	-0.38	2.33	lipoprotein NlpI; lipoprotein that appears to be involved in cell divis	Lipoprotein nlpI precursor
3050	STM14_39	STM14_39	CDS	pnp	3462431	3464596	-	-5.35	-6.20	0.01	-0.58	-0.40	5.80	Polyribonucleotide nucleotidyltransferase (EC 2.7.7.8); Polyadenylat	Polyribonucleotide nucleotidyltransferase (EC 2.7.7.8)
3058	STM14_39	STM14_39	CDS	secG	3475036	3475368	-	-1.80	-2.03	0.31	-0.33	-0.53	1.49	preprotein translocase subunit SecG; similar to Escherichia coli prot	Preprotein translocase subunit SecG (TC 3.A.5.1.1)
3060	STM3298	STM14_39	CDS	yhbY	3481337	3481669	+	-0.77	-2.32	0.25	1.87	5.17	7.49	RNA binding protein	FIG004454: RNA binding protein
3072	STM3313	STM14_40	CDS	yrbF	3492842	3493654	+	-7.29	-7.45	0.00	-0.86	-0.57	6.88	putative ABC transporter ATP-binding protein YrbF; ATP-binding sub	Uncharacterized ABC transporter, ATP-binding protein YrbF
3073	STM3314	STM14_40	CDS	yrbG	3493867	3494844	+	-3.84	-5.99	0.01	0.31	0.27	6.26	putative calcium/sodium:proton antiporter; YrbG; inner membrane	Inner membrane protein YrbG, predicted calcium/sodium:proton antipor
3102	STM3342	STM14_40	CDS	sspA	3524449	3525087	-	-1.93	-2.62	0.19	-0.17	-0.22	2.40	stringent starvation protein A; transcriptional activator; required fo	Stringent starvation protein A
3108	STM3350	STM14_40	CDS		3531877	3532608	-	-1.47	-2.75	0.17	0.79	1.72	4.47	putative inner membrane protein; similar to STM0063	Membrane protein associated with oxaloacetate decarboxylase
3114	STM3358	STM14_40	CDS		3539826	3540455	-	-1.14	-2.42	0.22	0.11	0.12	2.54	putative regulatory protein; GntR family	Transcriptional regulator, GntR family
3115	STM3359	STM14_40	CDS		3540466	3540597	-	-1.27	-2.04	0.31	-0.11	-0.15	1.89	malate dehydrogenase	Malate dehydrogenase (EC 1.1.1.37)
3183	STM3457	STM14_41	CDS	kefB	3621069	3622874	-	-0.71	-2.01	0.32	0.76	1.94	3.95	glutathione-regulated potassium-efflux system protein KefB; involve	Glutathione-regulated potassium-efflux system protein KefB
3188	STM3462	STM14_41	CDS	yheT	3626484	3627560	+	-0.81	-1.87	0.36	1.23	2.72	4.58	putative hydrolase. Alpha/beta-hydrolase fold	Hydrolase, alpha/beta fold family functionally coupled to Phosphoribulok
3216	STM3491	STM14_42	CDS	yrfB	3658652	3659125	-	-1.07	-3.16	0.12	1.05	2.35	5.51	putative inner membrane protein	Type IV pilus biogenesis protein PilO
3224	STM3499	STM14_42	CDS	yhgE	3668372	3670081	-	-1.17	-2.40	0.23	0.74	1.06	3.46	putative inner membrane protein; similar to Escherichia coli putativ	Putative transport protein
3241	STM3515	STM14_42	CDS	malT	3690798	3693503	+	-1.94	-2.84	0.16	-0.40	-0.33	2.50	transcriptional regulator MalT; Positively regulates the transcription	Transcriptional activator of maltose regulon, MalT
3243	STM3517	STM14_42	CDS		3693869	3694129	-	-7.18	-5.21	0.02	0.07	0.05	5.26	putative DNA-damage-inducibile protein; similar to Escherichia coli	DNA-damage-inducible protein J
3246	STM3521	STM14_42	CDS		3696653	3696871	+	-2.57	-3.09	0.13	0.77	1.40	4.49	hypothetical protein	
3247	STM3522	STM14_42	CDS	rtcR	3698566	3700149	+	-1.03	-2.46	0.22	0.23	0.31	2.77	sigma N; similar to Escherichia coli putative 2-component regulator	Transcriptional regulatory protein RtcR
3258	STM3533	STM14_42	CDS		3711678	3712433	-	-1.46	-2.77	0.17	0.27	0.23	3.00	putative transcriptional regulator; similar to Escherichia coli putativ	Transcriptional regulator, ArsR family
3262	STM3537	STM14_42	CDS	glgX_2	3717821	3719797	-	-1.23	-2.50	0.21	1.69	3.22	5.72	glycogen debranching enzyme; catalyzes the hydrolysis of the alpha	Glycogen debranching enzyme (EC 3.2.1.-)
3269	STM3543	STM14_42	CDS	gntR	3725866	3726861	-	-1.67	-2.42	0.22	0.33	0.68	3.11	gluconate operon transcriptional repressor; similar to Escherichia co	Gluconate utilization system Gnt-I transcriptional repressor
3289	STM3561	STM14_42	CDS	livG	3743067	3743834	-	-0.85	-2.09	0.30	1.65	3.65	5.74	leucine/isoleucine/valine transporter ATP-binding subunit; Part of th	Branched-chain amino acid transport ATP-binding protein LivG (TC 3.A.1.
3303	STM3577	STM14_43	CDS	tcp	3758540	3760183	+	-1.47	-3.52	0.09	1.49	3.45	6.97	methyl-accepting transmembrane citrate/phenol chemoreceptor; m	Methyl-accepting chemotaxis protein I (serine chemoreceptor protein)
3311	STM3585	STM14_43	CDS	yhhJ	3765532	3766656	-	-0.87	-1.76	0.39	2.14	4.69	6.45	putative ABC transport protein; similar to Escherichia coli putative t	FIG01045128: hypothetical protein
3320	STM3594	STM14_43	CDS	prlC	3777641	3779683	-	-3.36	-7.28	0.00	1.23	2.37	9.65	oligopeptidase A; oligopeptidase A (SW:OPDA_SALTY)	Oligopeptidase A (EC 3.4.24.70)
3322	STM3596	STM14_43	CDS	yhiR	3781347	3782189	+	-1.13	-1.55	0.46	1.29	2.42	3.97	putative cytoplasmic protein	Protein involved in catabolism of external DNA
3328	STM3602	STM14_43	CDS		3788230	3788949	-	-0.97	-2.63	0.19	0.29	0.47	3.10	putative regulatory protein; similar to Escherichia coli putative trans	Transcriptional regulator, GntR family
3355	STM3627	STM14_43	CDS	dppD	3825041	3826024	-	-1.44	-3.07	0.13	0.65	1.36	4.43	dipeptide transporter ATP-binding subunit; DppD and DppF are the	Dipeptide transport ATP-binding protein DppD (TC 3.A.1.5.2)
3361	STM3633	STM14_43	CDS		3832943	3833950	+	-0.80	-1.48	0.49	0.66	1.38	2.85	putative regulatory protein; similar to Escherichia coli regulator for	Putative lacI-family transcriptional regulator
3371	STM3643	STM14_43	CDS	yiaC	3843602	3844042	+	-1.43	-2.67	0.18	-0.07	-0.15	2.52	hypothetical protein	Acetyltransferase (EC 2.3.1.-)
3384	STM3657	STM14_44	CDS?		3856473	3856610	+	-0.87	-1.84	0.37	1.71	5.35	7.20	hypothetical protein	hypothetical protein
3408	STM3681	STM14_44	CDS		3883371	3884252	+	-1.67	-2.92	0.15	-0.08	-0.09	2.83	putative transcriptional regulator; similar to Escherichia coli possible	Putative sugar kinase
3428	STM3701	STM14_44	CDS	secB	3909728	3910195	-	-2.71	-3.08	0.13	-0.20	-0.24	2.84	preprotein translocase subunit SecB; molecular chaperone that is re	Protein export cytoplasm chaperone protein (SecB, maintains protein to
3431	STM3704	STM14_44	CDS	pmgI	3911256	3912800	+	-1.40	-2.56	0.20	0.82	2.17	4.72	phosphoglyceromutase; catalyzes the interconversion of 2-phospho	2,3-bisphosphoglycerate-independent phosphoglycerate mutase (EC 5.4.
3443	STM3718	STM14_44	CDS	rfaI	3927207	3928229	-	-1.17	-2.84	0.16	0.37	0.79	3.63	lipopolysaccharide 1,3-galactosyltransferase	UDP-glucose:(glucosyl)lipopolysaccharide alpha-1,3-glucosyltransferase V
3453	STM3733	STM14_44	CDS	pyrE	3939651	3940292	-	-1.12	-2.22	0.27	1.81	2.33	4.56	orotate phosphoribosyltransferase; involved in fifth step of pyrimidi	Orotate phosphoribosyltransferase (EC 2.4.2.10)
3460	STM3741	STM14_45	CDS	rpoZ	3947468	3947743	+	-2.58	-3.57	0.08	-0.50	-0.48	3.08	DNA-directed RNA polymerase subunit omega; promotes RNA polyr	DNA-directed RNA polymerase omega subunit (EC 2.7.7.6)
3482	STM3763	STM14_45	CDS	mgtB	3975234	3977960	-	-3.61	-7.41	0.00	-0.49	-0.44	6.97	Mg2+ transporter; Mg(2+) transport ATPase, P-type 2 (SW:ATMB_S/	Mg(2+) transport ATPase, P-type (EC 3.6.3.2)
3487	STM3768	STM14_45	CDS		3982236	3983345	-	-1.54	-2.96	0.14	-0.09	-0.23	2.73	putative selenocysteine synthase; L-seryl-tRNA selenium transferase	L-seryl-tRNA(Sec) selenium transferase-related protein
3491	STM3772	STM14_45	CDS		3985597	3986022	-	-1.01	-2.15	0.28	0.42	0.49	2.65	putative phosphotransferase system enzyme IIA	PTS system, mannose-specific IIA component

3493	STM3774	STM14_45	CDS		3989236	3989529	+	-2.44	-4.85	0.03	-0.05	-0.04	4.81	putative inner membrane protein	Putative secreted protein
3502	STM3783	STM14_45	CDS?		3997265	3997372	+	-3.80	-3.62	0.08	-0.04	-0.02	3.60	hypothetical protein	
3518	istR	STM14_45	CDS		4011777	4011950	+	-2.13	-1.96	0.33	1.11	1.20	3.16	hypothetical protein	hypothetical protein
3544	STM3828	STM14_46	CDS	dgoA	4044327	4045475	-	-0.84	-2.15	0.28	2.88	4.82	6.97	2-oxo-3-deoxygalactonate 6-phosphate aldolase/galactonate dehyd	Galactonate dehydratase (EC 4.2.1.6)
3545	STM14_46	STM14_46	CDS?	eda [J]	4045472	4046089	-	-1.78	-3.66	0.08	0.75	2.19	5.85	2-dehydro-3-deoxyphosphogalactonate aldolase (EC 4.1.2.21); D-ga	
3547	STM3830	STM14_46	CDS	dgoR	4046948	4047637	-	-1.87	-2.28	0.25	0.82	1.45	3.73	galactonate operon transcriptional repressor; similar to Escherichia	D-Galactonate repressor DgoR
3552	STM3836	STM14_46	CDS	recF	4054991	4056064	-	-1.74	-4.18	0.05	0.66	0.80	4.97	recombination protein F; Required for DNA replication; binds prefer	DNA recombination and repair protein RecF
3555	STM3841	STM14_46	CDS		4059858	4060115	+	-1.71	-3.79	0.07	0.69	0.65	4.44	hypothetical protein	Protein YidD
3561	STM3846.s	STM14_46	CDS		4066033	4066968	+	-5.59	-8.57	0.00	0.64	0.83	9.39	putative reverse transcriptase; RNA-dependent DNA polymerase	Retron-type RNA-directed DNA polymerase (EC 2.7.7.49)
3570	STM3856	STM14_46	CDS	pstC	4076183	4077142	-	-1.70	-2.04	0.31	0.99	1.16	3.21	phosphate transporter permease subunit; part of the ATP-depende	Phosphate transport system permease protein PstC (TC 3.A.1.7.1)
3582	STM3868	STM14_46	CDS	atpH	4091681	4092214	-	-2.34	-3.10	0.13	0.52	0.66	3.76	F0F1 ATP synthase subunit delta; Produces ATP from ADP in the pre	ATP synthase delta chain (EC 3.6.3.14)
3588	STM3874	STM14_46	CDS	gidA	4095591	4097480	-	-1.86	-2.65	0.19	-0.23	-0.40	2.24	tRNA uridine 5-carboxymethylaminomethyl modification enzyme Gi	tRNA uridine 5-carboxymethylaminomethyl modification enzyme GidA
3590	STM3876	STM14_46	CDS	asnC	4098392	4098850	-	-1.31	-1.73	0.41	0.47	0.55	2.28	DNA-binding transcriptional regulator AsnC; transcriptional repress	Regulatory protein AsnC
3595	STM3881	STM14_46	CDS	rbsD	4105352	4105771	+	-1.32	-3.45	0.09	1.18	1.25	4.70	D-ribose pyranase; cytoplasmic mutarotase that catalyzes the conve	Ribose ABC transport system, high affinity permease RbsD (TC 3.A.1.2.1)
3600	STM3886	STM14_46	CDS	rbsR	4110246	4111235	+	-2.41	-6.02	0.01	-0.27	-0.39	5.62	Ribose operon repressor; D-ribose utilization	Ribose operon repressor
3605	STM3900	STM14_46	CDS	ilvL	4122426	4122524	+	-1.09	-2.50	0.21	0.62	1.17	3.67	ilvG operon leader peptide; ilvGmedA operon leader peptide; atten	hypothetical protein
3625	STM3922	STM14_47	CDS	rffG	4145370	4146437	+	-0.63	-1.66	0.43	0.40	1.06	2.72	dTDP-glucose 4,6-dehydratase; similar to Escherichia coli dTDP-gluc	dTDP-glucose 4,6-dehydratase (EC 4.2.1.46)
3631	STM3929	STM14_47	CDS	wecG	4152164	4152904	+	-3.03	-2.44	0.22	-0.09	-0.09	2.35	putative UDP-N-acetyl-D-mannosaminuronic acid transferase; prob	Probable UDP-N-acetyl-D-mannosaminuronic acid transferase (EC 2.4.1.-
3634	STM3936	STM14_47	CDS	hemX	4156812	4157981	-	-2.22	-6.04	0.01	0.28	0.46	6.50	putative uroporphyrinogen III C-methyltransferase; similar to Esche	Homolog of E. coli HemX protein
3643	STM3946	STM14_47	CDS		4165641	4165973	-	-2.03	-4.74	0.03	0.15	0.14	4.88	hypothetical protein	
3644	STM3948	STM14_47	CDS	yigA	4166774	4167481	+	-1.08	-2.17	0.28	0.40	0.65	2.82	hypothetical protein; hypothetical protein in dapF-xerC intergenic r	Protein of unknown function DUF484
3664	STM3968	STM14_47	CDS	udp	4188555	4189316	+	-2.29	-4.75	0.03	0.93	1.75	6.50	uridine phosphorylase; catalyzes the reversible phosphorylytic clea	Uridine phosphorylase (EC 2.4.2.3)
3683	STM3995	STM14_48	CDS	yihD	4216434	4216703	+	-1.90	-3.18	0.12	0.69	1.12	4.30	putative cytoplasmic protein	Protein yihD
3685	STM3997	STM14_48	CDS	dsbA_2	4217783	4218406	+	-0.97	-2.45	0.22	1.52	1.93	4.38	periplasmic protein disulfide isomerase I; thiol-disulfide interchange	Periplasmic thiol:disulfide interchange protein DsbA
3701	STM4012	STM14_48	CDS		4234440	4235681	-	-2.47	-2.15	0.28	0.02	0.03	2.18	coproporphyrinogen III oxidase; catalyzes the oxygen-independent	Hypothetical radical SAM family enzyme in interesting gene cluster
3710	STM4021	STM14_48	CDS	manC [J]	4245190	4246431	-	-1.44	-3.11	0.12	0.37	0.95	4.06	putative isomerase	Aldose-ketose isomerase YihS
3727	STM14_48	STM14_48	CDS	fdoG	4258342	4261392	-	-1.14	-2.87	0.15	-0.01	-0.01	2.86	formate dehydrogenase alpha subunit; similar to Escherichia coli fo	Formate dehydrogenase O alpha subunit (EC 1.2.1.2) @ selenocysteine-c
3749	STM4058	STM14_48	CDS	cpxA	4281960	4283333	-	-3.03	-4.34	0.04	1.14	1.17	5.50	two-component sensor protein; part of two-component CpxA/CpxR	Copper sensory histidine kinase CpxA
3750	STM4059	STM14_48	CDS	cpxR	4283330	4284028	-	-0.86	-2.11	0.29	0.73	1.69	3.80	DNA-binding transcriptional regulator CpxR; response regulator in t	Copper-sensing two-component system response regulator CpxR
3756	STM4067	STM14_48	CDS	bvrC [J]_2	4291502	4292542	+	-2.46	-3.06	0.13	-0.60	-0.43	2.63	putative ADP-ribosylglycohydrolase	ADP-ribosylglycohydrolase (EC 3.2.-.-)
3768	STM4079.S	STM14_49	CDS	lsrG [R]	4302655	4302984	+	-1.85	-2.91	0.15	0.12	0.29	3.20	Autoinducer 2 (AI-2) modifying protein LsrG; Autoinducer 2 (AI-2) tr	Autoinducer 2 (AI-2) modifying protein LsrG
3770	STM4081	STM14_49	CDS		4303721	4304605	+	-1.21	-2.32	0.24	-0.11	-0.24	2.09	hypothetical protein	
3774	STM14_49	STM14_49	CDS	glpX	4306805	4307815	-	-1.04	-1.62	0.44	2.39	3.54	5.16	Fructose-1,6-bisphosphatase, GlpX type (EC 3.1.3.11); Glycolysis anc	Fructose-1,6-bisphosphatase, GlpX type (EC 3.1.3.11)
3779	STM4090	STM14_49	CDS	menA	4311739	4312668	-	-1.91	-5.18	0.02	1.60	2.70	7.88	1,4-dihydroxy-2-naphthoate octaprenyltransferase; catalyzes the fo	1,4-dihydroxy-2-naphthoate polyprenyltransferase (EC 2.5.1.74)
3789	STM14_49	STM14_49	CDS	metJ	4322716	4323129	-	-1.87	-1.73	0.41	-0.15	-0.18	1.55	transcriptional repressor of all met genes but metF (MetJ family)	Methionine repressor MetJ
3800	STM4110	STM14_49	CDS	ptsA	4338076	4340577	-	-1.37	-2.60	0.19	0.03	0.07	2.67	PEP-protein phosphotransferase; similar to Escherichia coli PEP-pro	Phosphoenolpyruvate-protein phosphotransferase of PTS system (EC 2.7.
3801	STM4111	STM14_49	CDS	ptsA	4338076	4340577	-	-1.79	-2.63	0.19	0.04	0.06	2.68	PEP-protein phosphotransferase; similar to Escherichia coli PEP-pro	Phosphoenolpyruvate-protein phosphotransferase of PTS system (EC 2.7.
3810	STM4120	STM14_49	CDS	argE	4351858	4353009	-	-1.75	-3.59	0.08	1.29	2.34	5.93	acetylornithine deacetylase; catalyzes the formation of L-ornithine f	Acetylornithine deacetylase (EC 3.5.1.16)
3817	STM4125	STM14_49	CDS	oxyR	4356665	4357582	+	-5.01	-10.76	0.00	0.62	1.72	12.48	DNA-binding transcriptional regulator OxyR; Activates the expressio	Hydrogen peroxide-inducible genes activator
3818	STM4127	STM14_49	CDS	fabR [R]	4359164	4359799	+	-2.15	-2.26	0.26	0.14	0.20	2.46	DNA-binding transcriptional repressor FabR; negatively controls the	Unsaturated fatty acid biosynthesis repressor FabR, TetR family
3825	STM4141	STM14_49	CDS		4373434	4373607	-	-3.09	-3.62	0.08	0.54	0.66	4.28	putative cytoplasmic protein	putative cytoplasmic protein
3831	STM4157	STM14_49	CDS		4388913	4389941	+	-1.99	-2.97	0.14	1.50	1.65	4.63	putative cytoplasmic protein	putative cytoplasmic protein
3839	STM4165	STM14_50	CDS	rsd	4396331	4396819	-	-2.46	-3.44	0.09	2.90	3.59	7.03	anti-RNA polymerase sigma 70 factor; binds specifically to the majo	Regulator of sigma D
3882	STM4215	STM14_50	CDS		4449116	4449571	-	-1.04	-2.50	0.21	0.13	0.29	2.79	putative cytoplasmic protein	Phage protein
3890	STM4223	STM14_50	CDS	yjbF	4456072	4456740	+	-1.46	-3.37	0.10	-0.24	-0.58	2.79	putative outer membrane lipoprotein	YjbF outer membrane lipoprotein
3891	STM4224	STM14_50	CDS	yjbG	4456737	4457474	+	-0.56	-1.57	0.46	0.42	1.04	2.61	putative periplasmic protein	YjbG polysaccharide synthesis-related protein
3901	STM4236	STM14_50	CDS	dgkA	4472313	4472681	+	-2.25	-2.50	0.21	1.38	1.13	3.63	diacylglycerol kinase; similar to Escherichia coli diacylglycerol kinase	Diacylglycerol kinase (EC 2.7.1.107)
3908	STM4244	STM14_51	CDS	pspG	4478668	4478910	+	-0.92	-2.71	0.18	1.41	1.86	4.57	phage shock protein G; coordinately regulated along with pspA; Psp	Phage shock protein G
3910	STM4247	STM14_51	CDS	alr	4481580	4482659	+	-3.41	-3.43	0.09	0.59	0.61	4.04	alanine racemase; converts L-alanine to D-alanine which is used in c	Alanine racemase (EC 5.1.1.1)
3925	STM4264	STM14_51	CDS	yjcC	4515902	4517524	+	-1.07	-2.11	0.29	0.53	0.71	2.82	GGDEF family protein	FIG00638940: hypothetical protein
3927	STM4266	STM14_51	CDS	soxR	4517922	4518380	+	-2.18	-5.00	0.02	1.67	1.90	6.90	redox-sensing transcriptional activator; redox-sensitive transcriptio	Redox-sensitive transcriptional activator SoxR
3943	STM4280	STM14_51	CDS	nrfD	4532425	4533381	+	-0.77	-1.48	0.49	0.98	1.81	3.29	putative formate-dependent nitrate reductase; similar to Escherichi	NrfD protein
3961	T9	STM14_51	sRNA_cand		4555760	4555856	+	-1.38	-3.24	0.11	-0.48	-0.45	2.79	DNA-binding transcriptional regulator MelR	
3995	STM4340	STM14_52	CDS	frdD	4595310	4595669	-	-1.34	-1.63	0.44	0.36	0.33	1.96	fumarate reductase subunit D; in conjunction with FrdC acts to anc	Fumarate reductase subunit D
3996	STM4341	STM14_52	CDS		4595377	4595862	+	-2.50	-3.02	0.13	1.57	3.34	6.36	hypothetical protein	
4003	STM4356	STM14_52	CDS	yjeF	4611198	4612745	+	-2.04	-3.76	0.07	1.17	1.69	5.45	hypothetical protein	NAD(P)HX epimerase / NAD(P)HX dehydratase
4012	STM4366	STM14_52	CDS	purA	4621926	4623224	+	-3.06	-2.79	0.17	0.26	0.26	3.05	adenylosuccinate synthetase; catalyzes the formation of N6-(1,2,-di	Adenylosuccinate synthetase (EC 6.3.4.4)
4029	STM4384	STM14_52	CDS		4639457	4639792	-	-1.90	-3.77	0.07	-0.17	-0.33	3.44	hypothetical protein	
4045	STM4404	STM14_52	CDS	cysQ	4654601	4655341	+	-1.41	-3.33	0.10	1.80	3.74	7.07	PAPS (adenosine 3'-phosphate 5'-phosphosulfate) 3'(2'),5'-bisphosp	3'(2'),5'-bisphosphate nucleotidase (EC 3.1.3.7)
4055	STM4415	STM14_53	CDS	fbp	4668328	4669326	-	-2.03	-2.79	0.17	0.87	0.97	3.76	fructose-1,6-bisphosphatase; catalyzes the formation of D-fructose	Fructose-1,6-bisphosphatase, type I (EC 3.1.3.11)
4080	STM4440	STM14_53	CDS		4696774	4697112	+	-1.51	-2.55	0.20	1.15	1.62	4.17	putative cytoplasmic protein; nitrogen regulation protein NR(ii) (SW	FIG01045571: hypothetical protein
4084	STM4444	STM14_53	CDS		4698598	4699242	+	-1.13	-3.04	0.13	0.68	1.93	4.97	putative inner membrane protein	Putative inner membrane protein
4086	STM4446	STM14_53	CDS		4700418	4701536	+	-0.93	-2.60	0.19	1.32	3.87	6.47	putative selenocysteine synthase; L-seryl-tRNA selenium transferase	D-Glucosaminate-6-phosphate ammonia-lyase (EC 4.3.1.-)
4088	STM4448	STM14_53	CDS		4702290	4704203	+	-2.59	-4.51	0.03	1.26	1.17	5.68	phosphotransferase system mannitol/fructose-specific IIA compone	Putative BglB-family transcriptional antiterminator
4089	STM4449	STM14_53	CDS	relB [R]	4704281	4704523	+	-2.09	-3.23	0.11	2.49	3.87	7.10	bifunctional antitoxin/transcriptional repressor RelB; Qin prophage;	RelB/StbD replicon stabilization protein (antitoxin to RelE/StbE)
4091	STM4451	STM14_53	CDS	nrdG	4704801	4705265	-	-5.40	-4.74	0.03	1.51	2.66	7.40	anaerobic ribonucleotide reductase-activating protein; activates an	Ribonucleotide reductase of class III (anaerobic), activating protein (EC 1.

4092	STM4452	STM14_53	CDS	nrdD	4705386	4707524	-	-4.47	-9.40	0.00	0.59	0.76	10.16	anaerobic ribonucleoside triphosphate reductase; Catalyzes the red	Ribonucleotide reductase of class III (anaerobic), large subunit (EC 1.17.4
4093	STM14_53	STM14_53	CDS		4707729	4707899	+	-1.94	-3.79	0.07	1.23	2.79	6.58	hypothetical protein	FIG01045722: hypothetical protein
4099	STM4458	STM14_53	CDS		4715791	4716264	+	-1.42	-2.11	0.29	0.28	0.34	2.46	hypothetical protein	
4102	STM4462	STM14_53	CDS	argR [R]	4717965	4718453	-	-4.90	-4.76	0.03	0.89	0.72	5.48	putative arginine repressor; similar to Escherichia coli repressor of a	Arginine pathway regulatory protein ArgR, repressor of arg regulon
4105	STM4465	STM14_53	CDS	argF [J]	4720099	4721103	-	-1.33	-1.51	0.48	1.25	1.80	3.31	ornithine carbamoyltransferase; catalyzes the formation of ornithin	Ornithine carbamoyltransferase (EC 2.1.3.3)
4107	STM4467	STM14_53	CDS	arcA [J]	4722158	4723378	-	-2.74	-6.72	0.00	-0.38	-0.45	6.27	arginine deiminase; catalyzes the degradation of arginine to citrulin	Arginine deiminase (EC 3.5.3.6)
4116	STM14_53	STM14_53	CDS	holC	4732701	4733183	-	-1.64	-1.91	0.35	-0.48	-0.32	1.59	DNA polymerase III, chi subunit	DNA polymerase III chi subunit (EC 2.7.7.7)
4128	STM4489	STM14_53	CDS		4744796	4748311	+	-1.44	-3.30	0.11	-0.06	-0.12	3.19	putative DNA helicase; similar to Escherichia coli putative frameshif	Putative superfamily I DNA helicases
4131	STM4492	STM14_53	CDS		4751605	4754208	-	-2.12	-5.27	0.02	0.03	0.06	5.34	putative cytoplasmic protein	putative cytoplasmic protein
4170	STM4527	STM14_54	CDS	mrr	4797299	4798213	+	-2.20	-3.53	0.09	0.18	0.46	3.99	restriction endonuclease; similar to Escherichia coli restriction of m	Mrr restriction system protein
4175	STM4532	STM14_54	CDS	cstA_2	4800398	4802548	-	-0.78	-1.86	0.36	2.50	3.58	5.44	putative carbon starvation protein; similar to Escherichia coli putati	Carbon starvation protein A paralog
4183	STM4540	STM14_54	CDS		4811388	4812398	+	-1.11	-2.75	0.17	0.24	0.45	3.20	putative glucosamine-fructose-6-phosphate aminotransferase; simil	Putative glucosamine-fructose-6-phosphate aminotransferase
4195	STM4557	STM14_54	CDS	holD	4825102	4825539	+	-4.06	-9.47	0.00	-0.05	-0.13	9.34	DNA polymerase III subunit psi; with the chi subunit binds to single-	DNA polymerase III psi subunit (EC 2.7.7.7)
4200	STM4561	STM14_54	CDS	osmY	4828712	4829329	+	-0.85	-1.47	0.50	0.07	0.06	1.53	periplasmic protein; similar to Escherichia coli hyperosmotically ind	Osmotically inducible protein OsmY
4204	STM4565	STM14_54	CDS	pflC [J]	4831619	4832482	-	-1.34	-3.01	0.13	1.57	2.67	5.68	pyruvate formate lyase-activating enzyme; similar to Escherichia col	radical activating enzyme
4216	STM4578	STM14_54	CDS	serB_2	4846057	4847025	+	-0.86	-2.15	0.28	0.06	0.09	2.24	phosphoserine phosphatase; catalyzes the formation of serine from	Phosphoserine phosphatase (EC 3.1.3.3)
4222	STM4585	STM14_55	CDS	gpmB	4854855	4855502	+	-2.64	-3.05	0.13	1.11	1.89	4.94	phosphoglycerate mutase; catalyzes reactions involving the transfe	Phosphoglycerate mutase (EC 5.4.2.1)
4225	STM4588	STM14_55	CDS	creB	4857066	4857755	+	-1.25	-2.90	0.15	-0.14	-0.21	2.69	DNA-binding response regulator CreB; response regulator in two-co	Two-component response regulator CreB
4241	PSLT004	STM14_55	CDS	repA3	1722	1844	+	-1.44	-1.98	0.32	0.56	0.74	2.72	DNA replication	hypothetical protein
4242	PSLT005	STM14_55	CDS	repA	1907	2788	+	-1.29	-1.70	0.42	0.56	0.76	2.46	DNA replication	DNA replication protein
4297	PSLT059	STM14_55	CDS		49478	50158	+	-1.67	-2.63	0.19	2.05	3.30	5.93	putative adenine-specific DNA methylase	Adenine-specific methyltransferase (EC 2.1.1.72)
4298	PSLT060	STM14_55	CDS		50158	50379	+	-2.23	-3.18	0.12	0.92	1.14	4.33	putative cytoplasmic protein	putative cytoplasmic protein
4330	PSLT094	STM14_56	CDS	trbC	73506	74144	+	-0.71	-1.59	0.45	0.81	2.18	3.76	conjugative transfer: assembly	IncF plasmid conjugative transfer protein TrbC
4335	PSLT098	STM14_56	CDS	traQ	77624	77917	+	-1.08	-2.19	0.27	-0.23	-0.36	1.83	conjugative transfer: fimbrial synthesis	IncF plasmid conjugative transfer protein TraQ
4343	STM14_56	STM14_56	CDS	trbH	86459	87178	+	-1.11	-2.77	0.17	0.90	2.15	4.92	conjugative transfer protein	IncF plasmid conjugative transfer protein TrbH
4345	PSLT110	STM14_56	CDS	traX	92453	93193	+	-0.92	-2.06	0.30	0.85	2.26	4.32	conjugative transfer: fimbrial acetylation	IncF plasmid conjugative transfer pilin acetylase TraX

1.5.5.-)

:eine nucleosidase (EC 3.2.2.9)

5.3.5); Copper-translocating P-type ATPase (EC 3.6.3.4)

required for thiamine synthesis

ophore biosynthesis

dehydrogenase complex (EC 2.3.1.61)

etabolism

or components CheY
(EC 3.1.1.61)

nsporter

r HisJ (TC 3.A.1.3.1)

embly complex (forms a complex with YaeT, YfiO, and NlpB)

C components (EC 2.7.1.69)
asmic component

)

-regulated ECF transporter

15)

midohydrolase (EC 3.5.1.78)

ıt

ter

:inase

4.1)

be exported in unfolded state)
2.1)
WaaO (EC 2.4.1.-)

)

ontaining

.3.9)
.3.9)

97.1.4)

.2)
