# Supplementary material for: Solid tumors provide niche-specific conditions that lead to preferential growth of Salmonella
Source: Oncotarget. 2016 Apr 28;7(23):35169–80. doi: 10.18632/oncotarget.9071 (PMC5085218; doi:10.18632/oncotarget.9071)
Supplement: Supplementary file 3 [file oncotarget-07-35169-s003.pdf]

Supplementary Table S2. Mutants more fit in tumors but not more fit in spleen.

Data are color coded on an arbitrary scale to represent the direction and amount of change

A partial list of arbitrary cutoff thresholds for generating the list include a log2 fold change of >0.5 in tumor, T value >1.4 in tumor, and an FDR <0.5 in tumor. Difference between spleen and tumor T value of <-1.4.

Gene order on genome	Gene	14028_gene	Gene_Type	Gene_name	start	end	strand	tumor<br>Log2 Fold change versus input	tumor<br>t value	tumor<br>False Discovery Rate	spleen<br>Log2 Fold change versus input	spleen<br>t value	speen t value minus tumor t value	final_function	RAST Function
2	ryaA	0	0		0	0	0	1.24	2.83	0.16	0.16	0.40	-2.43		
33	STM0029	STM14_0037	CDS	marT [J]_2	32545	32994	-	0.68	1.81	0.38	-2.61	-4.34	-6.15	putative transcriptional regulator; putative trar	Putative membrane protein
36	STM0032	STM14_0041	CDS		35339	37057	+	1.83	2.27	0.26	-1.84	-1.36	-3.63	putative arylsulfatase	Possible sulfatase
101	STM0100	STM14_0119	CDS		117528	117947	+	1.86	4.87	0.03	0.04	0.07	-4.81	putative cytoplasmic protein	FIG01045455: hypothetical protein
114	STM0111	STM14_0132	CDS	leuC	130385	131785	-	1.16	2.56	0.20	-0.45	-0.86	-3.42	isopropylmalate isomerase large subunit; dehy	3-isopropylmalate dehydratase large subunit (EC 4.2.1.33)
116	STM0113	STM14_0134	CDS	leuA	132879	134450	-	1.45	3.06	0.13	-0.52	-0.87	-3.93	2-isopropylmalate synthase; catalyzes the form	2-isopropylmalate synthase (EC 2.3.3.13)
172	STM0180	STM14_0213	CDS	panD	211735	212115	-	1.06	1.86	0.36	-0.34	-0.46	-2.31	aspartate alpha-decarboxylase; Converts L-asp	Aspartate 1-decarboxylase (EC 4.1.1.11)
194	STM0203	STM14_0243	CDS	clcA	239365	240786	+	1.46	2.81	0.16	-0.31	-0.47	-3.28	chloride channel protein; Acts as an electrical s	H(+)/Cl(-) exchange transporter ClcA
224	STM0244	STM14_0286	CDS	rcsF	285700	286104	-	1.39	2.66	0.18	-0.21	-0.31	-2.97	outer membrane lipoprotein; similar to Escheri	Protein RcsF
267	STM0294.1N	STM14_0346	CDS		340013	340288	-	3.32	4.85	0.03	-0.64	-0.84	-5.70	hypothetical protein	FIG01046370: hypothetical protein
285	STM0312	STM14_0367	CDS	yafJ	356757	357524	+	3.05	7.18	0.00	-0.07	-0.17	-7.36	putative glutamine amidotransferase; similar tc	Predicted glutamine amidotransferase
289	STM0316	STM14_0372	CDS	pepD	361658	363115	-	2.11	3.07	0.13	0.01	0.01	-3.06	aminoacyl-histidine dipeptidase; similar to Esch	Aminoacyl-histidine dipeptidase (Peptidase D) (EC 3.4.13.3
299	STM0327	STM14_0383	CDS/sRNA?		371424	371747	+	1.41	3.55	0.08	-1.18	-2.16	-5.70	putative cytoplasmic protein	FIG074102: hypothetical protein
317	STM0345	STM14_0402	CDS		390191	390679	+	0.63	1.69	0.42	-6.00	-7.93	-9.62	putative inner membrane protein	Putative inner membrane protein
338	STM0366	STM14_0428	CDS	yahO	416369	416644	+	1.74	1.84	0.37	-1.51	-1.87	-3.71	hypothetical protein	FIG00639237: hypothetical protein
364	STM0393	STM14_0465	CDS	yajF	444659	445567	+	2.12	5.14	0.02	-0.34	-0.56	-5.69	fructokinase; catalyzes phosphorylation of fruc	Glucokinase, ROK family (EC 2.7.1.2)
392	STM0426	STM14_0504	CDS	phnV	480053	480850	-	2.55	3.29	0.11	0.64	0.44	-2.85	2-aminoethylphosphonate transporter; probab	2-aminoethylphosphonate ABC transporter permease prot
426	STM0457	STM14_0541	CDS	cof	514925	515743	+	2.68	3.26	0.11	0.28	0.25	-3.01	putative hydrolase	HMP-PP hydrolase (pyridoxal phosphatase) Cof, detected i
430	STM0461	STM14_0545	CDS	mdlB	519224	521005	+	2.48	3.34	0.10	0.31	0.41	-2.93	putative multidrug transporter membrane\ATP	putative multidrug transporter membrane ATP-binding cor
436	STM0466	STM14_0550	CDS	ybaZ	524651	525040	-	1.34	1.71	0.42	-1.13	-0.87	-2.58	putative methyltransferase	methylated-DNA--protein-cysteine methyltransferase-relat
447	STM0477	STM14_0561	CDS	acrR	534448	535101	+	1.45	3.50	0.09	-0.56	-1.02	-4.51	DNA-binding transcriptional repressor AcrR; reg	Transcription repressor of multidrug efflux pump acrAB op
448	STM14_0563.J	STM14_0563.J	CDS	aefA	535160	538582	+	2.03	3.57	0.08	-0.61	-0.57	-4.14	mechanosensitive channel protein	Potassium efflux system KefA protein / Small-conductance
516	STM0549	STM14_0641.J	CDS	fimZ	610950	611675	-	1.70	4.15	0.05	-0.87	-1.15	-5.30	fimbriae z protein. {salmonella	Transcriptional regulator of fimbriae expression FimZ (LuxR
531	STM0565	STM14_0660	CDS		622504	622740	+	1.89	2.61	0.19	-3.11	-2.50	-5.11	putative periplasmic protein	Putative periplasmic protein
534	STM0568	STM14_0663	CDS	pheP	624893	626287	+	1.83	2.91	0.15	-1.40	-1.61	-4.51	phenylalanine transporter; similar to Escherichi	Phenylalanine-specific permease
578	STM0613	STM14_0712	CDS	dmsC [J]_1	677033	677800	+	1.84	2.72	0.17	-0.14	-0.18	-2.91	putative hydrogenase protein; similar to Escher	Anaerobic dimethyl sulfoxide reductase chain C (EC 1.8.5.3
622	STM0662	STM14_0770	CDS	gltL	727107	727832	-	1.16	2.15	0.28	-0.11	-0.16	-2.31	glutamate/aspartate transporter; similar to Esc	Glutamate Aspartate transport ATP-binding protein GltL (T
644	STM0691	STM14_0806	CDS	tcuA [R]	754349	755752	-	1.12	3.06	0.13	0.13	0.20	-2.86	tricarballylate dehydrogenase; catalyzes the ox	TcuA: flavoprotein used to oxidize tricarballylate to cis-aco
659	STM0708	STM14_0827	CDS	ybfA	774958	775164	+	0.81	1.52	0.48	-0.52	-0.79	-2.31	putative periplasmic protein	FIG01200701: possible membrane protein
661	STM0710	STM14_0829	CDS	ybgH	776735	778330	-	1.31	2.86	0.16	0.22	0.46	-2.40	POT family transport protein; similar to Escheri	Di/tripeptide permease YjdL
665	STM0714	STM14_0833	CDS	ybgL	780876	781610	+	0.73	1.45	0.50	-1.97	-1.61	-3.06	hypothetical protein; similar to Escherichia coli	Lactam utilization protein LamB
676	STM0725	STM14_0844	CDS		791382	792215	+	1.81	2.51	0.21	0.09	0.10	-2.41	putative glycosyltransferase; cell wall biogenesi	FIG01045205: hypothetical protein
686	STM0735	STM14_0854	CDS	sdhB	800999	801718	+	1.86	4.01	0.05	-3.36	-2.95	-6.97	succinate dehydrogenase iron-sulfur subunit; p	Succinate dehydrogenase iron-sulfur protein (EC 1.3.99.1)
708	STM0763	STM14_0887	CDS		826795	827685	+	0.61	1.64	0.44	-5.03	-5.65	-7.29	transcriptional regulator; similar to Escherichia	Transcriptional regulator, lysR family
709	STM0764	STM14_0888	CDS		827686	828669	-	2.09	2.92	0.15	0.22	0.40	-2.53	transcriptional regulator; similar to Escherichia	LysR-family transcriptional regulator STM0764
743	STM14_0926.L	STM14_0926.L	?		866881	867036	+	0.91	2.39	0.23	-3.93	-5.37	-7.75		
777	STM0833	STM14_0971.J	CDS	ompX	900580	901101	+	1.08	2.35	0.24	-1.80	-1.87	-4.21	outer membrane protease, receptor for phage	Outer membrane protein X precursor
795	STM0849	STM14_0992	CDS	yliB	921057	922595	+	1.47	3.28	0.11	0.14	0.24	-3.04	putative ABC transporter periplasmic binding p	Dipeptide transport system, periplasmic component in pro
829	STM0881	STM14_1032.RJ	CDS	ybjO	953559	954122	+	1.08	2.59	0.19	-0.38	-0.85	-3.44	Putative inner membrane protein	Putative inner membrane protein
834	STM0887	STM14_1039	CDS	artJ	958987	959718	-	0.94	2.66	0.18	-0.09	-0.21	-2.87	arginine transport system component; similar t	Arginine ABC transporter, periplasmic arginine-binding pro
879	STM0975	STM14_1101	CDS	ycaO	1017059	1018819	-	1.36	2.98	0.14	-2.49	-3.14	-6.12	putative cytoplasmic protein	FIG00638589: hypothetical protein
894	STM0996	STM14_1126	CDS	ycbK	1045480	1046028	+	1.33	2.74	0.17	-1.35	-2.73	-5.48	putative outer membrane protein	FIG001587: exported protein
895	STM0997	STM14_1127	CDS	ycbL	1046056	1046703	+	1.38	3.11	0.12	-0.15	-0.24	-3.36	putative metallo-beta-lactamase	Hypothetical metal-binding enzyme, YcbL homolog
914	STM1023	STM14_1163	CDS		1070732	1071049	+	1.44	4.07	0.05	0.18	0.16	-3.90	hypothetical protein	Phage protein
934	STM1043	STM14_1183	CDS		1087783	1088280	+	0.93	1.72	0.41	-1.01	-0.83	-2.55	attachment/invasion protein; similar to OmpX (	Attachment invasion locus protein precursor

968	STM1077	STM14_1222	CDS	yccT	1127421	1128083	-	1.34	3.14	0.12	-0.66	-1.29	-4.43	hypothetical protein	UPF0319 protein YccT precursor
980	STM1090	STM14_1236	CDS	pipC	1136217	1136558	-	1.03	2.16	0.28	-4.77	-8.07	-10.23	pathogenicity island-encoded protein C; similar	Invasion gene E protein (Pathogenicity island encoded prot
984	STM1094	STM14_1240	CDS	pipD	1138982	1140544	-	1.90	2.80	0.17	-5.73	-5.16	-7.96	pathogenicity island-encoded protein D; similar	Probable dipeptidase (EC 3.4.-.-)
1001	STM1111	STM14_1263	CDS	cbpM	1157038	1157343	-	1.28	3.33	0.10	-2.00	-2.13	-5.46	chaperone-modulator protein CbpM; with Cpb/	Chaperone-modulator protein CbpM
1034	STM1144	STM14_1310	CDS	csgA	1189792	1190247	+	0.58	1.57	0.46	-3.43	-3.69	-5.26	cryptic curlin major subunit; major curlin subun	Major curlin subunit precursor CsgA
1046	STM1156	STM14_1324	CDS	yceA	1201918	1202970	+	1.80	2.55	0.20	-0.19	-0.29	-2.84	hypothetical protein	Rhodanese domain protein, Enterobacterial subgroup, Ycea
1050	STM1160	STM14_1329	CDS	solA	1204583	1205701	-	1.29	2.39	0.23	-2.95	-3.06	-5.45	N-methyltryptophan oxidase; catalyzes the den	N-methyl-L-amino-acid oxidase (EC 1.5.3.2); N-methyl-L-try
1089	STM1203	STM14_1377	CDS	ptsG	1244532	1245965	+	1.65	2.84	0.16	-0.50	-0.61	-3.45	glucose-specific PTS system IIBC components; p	PTS system, glucose-specific IIB component (EC 2.7.1.69) /
1137	STM14_1491	STM14_1491	cDNA/sRNA?	IsrC [P]	1339203	1339362	+	1.97	2.45	0.22	-0.45	-0.59	-3.04		
1159	STM1263	STM14_1523	CDS	zinT [R]	1355815	1356462	+	1.07	1.70	0.42	-1.47	-1.46	-3.16	hypothetical protein; similar to C-terminus of a	Candidate zinc-binding lipoprotein ZinT
1169	STM1273	STM14_1537	CDS		1362158	1362799	+	1.30	2.18	0.28	-0.59	-0.76	-2.94	putative nitric oxide reductase	Putative membrane protein
1196	STM1299	STM14_1577	CDS	gdhA	1388612	1389955	-	1.47	2.76	0.17	-0.50	-0.55	-3.31	glutamate dehydrogenase; converts 2-oxogluta	NADP-specific glutamate dehydrogenase (EC 1.4.1.4)
1217	STM1320	STM14_1604	CDS	gltP [J]_1	1410051	1411442	-	1.52	3.43	0.09	0.26	0.41	-3.01	kinase/transporter-like protein; similar to Esche	L-cystine uptake protein TcyP
1225	STM1328	STM14_1612	CDS		1417094	1418053	-	2.79	3.51	0.09	0.41	0.27	-3.24	putative outer membrane protein	putative outer membrane protein
1245	STM1349	STM14_1639	CDS	pps	1437746	1440124	+	2.08	3.46	0.09	-0.89	-0.98	-4.44	phosphoenolpyruvate synthase; catalyzes the f	Phosphoenolpyruvate synthase (EC 2.7.9.2)
1251	STM1355	STM14_1646	CDS	ydiP	1445713	1446603	+	1.22	3.32	0.10	-1.35	-2.00	-5.31	putative transcriptional regulator; similar to Es	Transcriptional regulator, AraC family
1261	STM1364	STM14_1657	CDS	ydiK	1455097	1456215	-	1.92	2.89	0.15	0.44	0.39	-2.50	putative inner membrane protein; YdiK; inner m	Putative membrane protein
1273	STM1374	STM14_1668	CDS	sufE_1	1467394	1467810	+	2.04	3.66	0.08	-0.40	-0.54	-4.21	cysteine desufuration protein SufE; Acts with Su	Sulfur acceptor protein SufE for iron-sulfur cluster assembl
1274	STM1375	STM14_1669	CDS	srfK [D]_2	1467965	1468966	+	2.58	7.21	0.00	-0.51	-0.95	-8.16	hypothetical protein; contains putative LysM do	L,D-transpeptidase YnhG
1281	STM1382	STM14_1676	CDS	orf408	1474782	1476008	-	0.89	1.58	0.45	-0.92	-1.03	-2.61	putative regulatory protein; ORF 408 (gi|44568	Ribokinase (EC 2.7.1.15)
1323	STM1427	STM14_1724	CDS	cfa	1514211	1515359	-	1.71	3.89	0.06	-0.74	-1.49	-5.38	cyclopropane fatty acyl phospholipid synthase;	Cyclopropane-fatty-acyl-phospholipid synthase (EC 2.1.1.79
1331	STM1435	STM14_1732.J	CDS?		1522308	1522457	+	1.13	1.63	0.44	-0.73	-1.06	-2.69	hypothetical protein	
1341	STM1445	STM14_1743	CDS	slyB	1530344	1530811	-	1.26	1.63	0.44	-0.86	-0.75	-2.38	putative outer membrane lipoprotein; outer me	Outer membrane lipoprotein pcp precursor
1344	STM1450	STM14_1748	CDS	pdxY	1534797	1535657	+	2.37	2.75	0.17	-0.32	-0.31	-3.07	pyridoxamine kinase; catalyzes the formation o	Pyridoxal kinase (EC 2.7.1.35)
1355	STM14_1760.J	STM14_1760.J	CDS	hha [J]	1545511	1545795	-	0.89	2.24	0.26	-0.46	-0.79	-3.03	putative cytoplasmic protein	Cnu protein
1370	STM1476	STM14_1780	CDS	glpM	1562124	1562459	+	2.79	3.02	0.13	-0.06	-0.07	-3.09	putative inner membrane protein	Putative inner membrane protein
1378	STM1484	STM14_1793	CDS		1570946	1571767	-	1.08	2.54	0.20	-1.49	-2.55	-5.09	putative protease	FIG00634494: possible peptidase
1380	STM1486	STM14_1797	CDS	ynfM	1572679	1573932	-	1.37	3.03	0.13	-2.33	-3.82	-6.85	putative transport protein; similar to Escherichi	Permeases of the major facilitator superfamily
1385	STM1491	STM14_1802	CDS	opuBA [J]	1578501	1579649	-	1.32	3.10	0.13	-1.28	-1.85	-4.94	proline/glycine betaine transport system; simila	L-proline glycine betaine ABC transport system permease p
1400	STM1508	STM14_1822	CDS	mtlK [J]_1	1596047	1597513	+	2.43	4.57	0.03	-0.66	-0.92	-5.49	putative mannitol dehydrogenase; similar to Es	D-mannonate oxidoreductase (EC 1.1.1.57)
1404	STM1512	STM14_1826	CDS	dcp	1599665	1601707	+	1.79	4.24	0.05	-0.10	-0.10	-4.35	dipeptidyl carboxypeptidase II; peptidyl-dipepti	Dipeptidyl carboxypeptidase Dcp (EC 3.4.15.5)
1420	STM1528	STM14_1846	CDS		1615133	1615675	-	3.31	3.83	0.07	0.31	0.28	-3.55	putative outer membrane protein	putative outer membrane protein
1433	STM1541	STM14_1860	CDS		1626447	1627238	+	2.30	3.00	0.14	-0.94	-0.93	-3.93	putative regulatory protein; similar to Escherich	Hexuronate utilization operon transcriptional repressor Exu
1440	STM1548	STM14_1868	CDS	queA [J]	1634851	1635930	+	1.26	2.67	0.18	-2.18	-3.17	-5.84	putative S-adenosylmethionine/tRNA-ribosyltra	S-adenosylmethionine:tRNA ribosyltransferase-isomerase (
1447	STM1554	STM14_1877	CDS		1640382	1641848	-	1.33	2.23	0.26	0.35	0.50	-1.73	putative coiled-coil protein	putative coiled-coil protein
1449	STM1556	STM14_1879	CDS	nhaC	1643258	1644709	+	1.08	2.49	0.21	-1.20	-1.76	-4.24	putative Na+/H+ antiporter; similar to Escherich	FIG01045761: hypothetical protein
1458	STM1565	STM14_1888	CDS	rpsV	1655468	1655611	+	1.59	2.62	0.19	-1.58	-1.92	-4.54	30S ribosomal subunit S22; protein D; stationar	Stationary-phase-induced ribosome-associated protein
1461	STM1568	STM14_1892	CDS	fdnI	1658872	1659528	-	1.58	3.20	0.12	0.29	0.35	-2.84	formate dehydrogenase-N subunit gamma; nitr	Formate dehydrogenase N gamma subunit (EC 1.2.1.2)
1524	STM1631	STM14_1974	CDS	sseJ	1731270	1732496	+	2.19	3.86	0.06	-5.02	-7.04	-10.90	secreted effector protein; secreted effector J (g	secreted effector protein
1534	STM1639	STM14_1983	CDS	cybB	1739800	1740330	-	1.99	3.01	0.13	0.23	0.20	-2.82	cytochrome b561; B-type di-heme cytochrome	Cytochrome b(561)
1537	STM1642	STM14_1986	CDS	acpD	1745414	1746019	+	0.92	2.03	0.31	-0.06	-0.21	-2.24	azoreductase; FMN-dependent; requires NADH	FMN-dependent NADH-azoreductase
1550	STM1653	STM14_1998	CDS	emrE	1756592	1756930	-	3.01	4.08	0.05	-0.64	-0.47	-4.56	putative membrane transporter of cations; sim	Ethidium bromide-methyl viologen resistance protein EmrE
1575	STM1676	STM14_2023	CDS		1778782	1779651	-	1.18	2.33	0.24	0.08	0.08	-2.25	putative aldo/keto reductase	oxidoreductase, aldo/keto reductase family
1585	STM1687	STM14_2037	CDS	pspD	1791553	1791771	-	2.73	3.29	0.11	-1.41	-1.45	-4.74	peripheral inner membrane phage-shock prote	Phage shock protein D
1586	STM1688	STM14_2038	CDS	pspC	1791794	1792153	-	1.33	2.74	0.17	-1.60	-1.43	-4.17	DNA-binding transcriptional activator PspC; wit	Phage shock protein C
1618	STM1722	STM14_2084.J	CDS?		1829078	1829248	-	1.34	2.89	0.15	-0.35	-0.34	-3.24	hypothetical protein	
1624	STM1728	STM14_2091	CDS	yciG	1836247	1836429	+	1.48	1.89	0.35	-0.17	-0.24	-2.12	putative cytoplasmic protein	Conidiation-specific protein 10
1641	STM1745	STM14_2109	CDS	oppB_1	1849255	1850175	-	2.05	5.05	0.02	-0.05	-0.10	-5.15	oligopeptide transporter permease; oligopeptic	Oligopeptide transport system permease protein OppB (TC
1642	STM1746	STM14_2109.J	CDS?		1850195	1850317	+	1.24	2.84	0.16	-1.63	-3.25	-6.09	hypothetical protein	
1663	STM1769	STM14_2139	CDS	ychN	1879012	1879365	+	1.88	3.22	0.11	-0.55	-0.68	-3.90	putative sulfur reduction protein; putative ACR	Putative ACR protein
1680	STM1788	STM14_2163	CDS	hyaC [D]	1898444	1899175	+	1.69	3.79	0.07	0.05	0.09	-3.70	hydrogenase 1 b-type cytochrome subunit; sim	Ni,Fe-hydrogenase I cytochrome b subunit
1681	STM1789	STM14_2164	CDS		1899172	1899768	+	1.67	3.69	0.07	-0.30	-0.46	-4.15	hydrogenase 1 maturation protease; HyaD; enc	Hydrogenase maturation protease (EC 3.4.24.-)
1688	STM1796	STM14_2172	CDS	treA	1905759	1907471	+	1.66	4.45	0.04	-0.65	-1.27	-5.73	trehalase; periplasmic; catalyzes the hydrolysis	Trehalase (EC 3.2.1.28); Periplasmic trehalase precursor (E
1826	STM1927	STM14_2344	CDS	yecG	2034258	2034686	+	1.63	3.02	0.13	-1.00	-0.92	-3.95	universal stress protein UspC; ppGpp-depender	Universal stress protein C
1829	STM1930	STM14_2347	pseudo		2037066	2037308	-	0.89	1.62	0.44	0.04	0.06	-1.56	pseudogene; frameshift	
1846	STM1951	STM14_2369	CDS	glnQ [J]	2052017	2052769	-	1.67	2.72	0.17	-3.50	-7.45	-10.17	putative amino-acid ABC transporter ATP-bindi	Cystine ABC transporter, ATP-binding protein
1849	STM1954	STM14_2372	CDS	fliY	2054595	2055395	-	1.65	2.62	0.19	0.10	0.10	-2.52	cystine transporter subunit; similar to Escherich	Cystine ABC transporter, periplasmic cystine-binding prote
1861	STM1967	STM14_2387.J	CDS		2066904	2067293	+	1.42	2.58	0.19	-2.70	-5.35	-7.94	putative 50S ribosomal protein	FIG01200701: possible membrane protein
2030	STM2135	STM14_2633	CDS		2279010	2281121	+	1.41	3.03	0.13	-1.19	-2.08	-5.11	putative inner membrane protein	Putative inner membrane protein
2033	STM2137	STM14_2636	pseudo		2283049	2284095	+	1.84	2.40	0.23	0.44	0.36	-2.04	putative cytoplasmic protein~disrupted by fran	
2059	STM2162	STM14_2665	CDS		2308712	2308819	+	3.16	4.04	0.05	-0.09	-0.08	-4.12	hypothetical protein	hypothetical protein

2070	STM2173	STM14_2680	CDS		2322009	2322206	+	1.08	1.66	0.43	-1.07	-1.34	-2.99	conserved hypothetical protein	FIG01045646: hypothetical protein
2080	STM2183	STM14_2690	CDS	cdd	2330719	2331603	+	0.92	2.33	0.24	-1.51	-1.63	-3.96	cytidine deaminase; Reclaims exogenous and e	Cytidine deaminase (EC 3.5.4.5)
2101	STM2204	STM14_2721	CDS	fruA	2355134	2356822	-	1.28	1.81	0.38	-0.16	-0.16	-1.97	fructose-specific PTS system IIBC component; p	PTS system, fructose-specific IIB component (EC 2.7.1.69) /
2119	STM2221	STM14_2745	CDS		2373810	2374100	-	1.36	1.58	0.45	-0.05	-0.09	-1.67	bicyclomycin/multidrug efflux system	sensor histidine kinase
2123	STM2225	STM14_2751	CDS		2378379	2378999	-	1.56	2.67	0.18	0.06	0.07	-2.60	putative inner membrane protein	Putative inner membrane protein
2143	STM2251STM38	STM14_2780	CDS	ccmD_1	2402944	2403156	-	1.66	2.86	0.16	-0.12	-0.14	-3.00	heme exporter protein C; similar to Escherichia	Cytochrome c-type biogenesis protein CcmD, interacts witl
2185	STM2295	STM14_2832	CDS	yfaO	2454150	2454575	+	1.84	3.17	0.12	-0.26	-0.27	-3.44	putative NTP pyrophosphohydrolase; similar to	Pyrimidine deoxynucleoside triphosphate (dYTP) pyrophos
2226	STM2336	STM14_2879	CDS		2498696	2499151	-	1.26	2.76	0.17	-0.38	-0.68	-3.43	hypothetical protein	FIG00638298: membrane protein YfbV
2234	STM2344	STM14_2889	CDS		2508239	2508682	-	0.81	1.94	0.33	-4.13	-5.36	-7.30	putative phosphotransferase system enzyme II	Ascorbate-specific PTS system, EIIA component (EC 2.7.1.-)
2265	STM2376	STM14_2925	CDS		2538835	2539353	+	1.02	2.55	0.20	-6.17	-7.98	-10.53	putative periplasmic protein	FIG00545237: hypothetical protein
2288	STM2400	STM14_2951	CDS		2565349	2565591	-	1.47	2.44	0.22	-1.32	-2.48	-4.92	putative inner membrane protein	FIG00638164: hypothetical protein
2304	STM2422	STM14_2977.RJ	CDS	deoD [J]	2586410	2587243	-	0.84	1.99	0.32	-4.44	-4.47	-6.46	Xanthosine phosphorylase (EC 2.4.2.1); Xantho:	Xanthosine phosphorylase (EC 2.4.2.1)
2305	STM2423	STM14_2978	CDS	yfeN	2587499	2588260	+	3.34	4.60	0.03	0.37	0.45	-4.15	hypothetical protein; similar to Escherichia coli	Putative exported protein precursor
2354	STM2475	STM14_3035	CDS		2637115	2637345	-	2.56	2.62	0.19	-0.01	-0.01	-2.63	putative cytoplasmic protein	FIG01046050: hypothetical protein
2405	STM2527	STM14_3100	CDS		2712174	2713037	-	1.08	2.44	0.22	-0.44	-0.91	-3.35	putative polyferredoxin; similar to Escherichia c	Putative polyferredoxin
2409	STM2531	STM14_3104	CDS	pbpC	2716999	2719314	-	1.36	1.89	0.35	-1.12	-1.05	-2.94	penicillin-binding protein 1C; penicillin-insensiti	Penicillin-insensitive transglycosylase (EC 2.4.2.-) & transpe
2416	STM2537	STM14_3112	CDS	iscX [D]	2729200	2729400	-	1.20	2.84	0.16	-0.78	-1.51	-4.35	hypothetical protein; believed to be involved in	Believed to be involved in assembly of Fe-S clusters
2424	STM2547	STM14_3125	CDS	yfhR [R]	2736936	2737814	+	0.72	2.03	0.31	0.26	0.30	-1.73	putative hydrolase; similar to Escherichia coli p	Uncharacterized protein yfhR
2478	STM2645	STM14_3242	CDS	yfiK	2839091	2839678	+	2.35	2.68	0.18	-1.38	-1.32	-4.00	neutral amino-acid efflux protein	Transporter, LysE family
2526	STM2746	STM14_3310	CDS		2906298	2907581	+	1.00	2.03	0.31	-6.90	-9.04	-11.07	putative ATPase; excinuclease	putative ATPase
2534	STM2754	STM14_3319	CDS		2912634	2913836	+	1.21	1.84	0.37	-0.21	-0.33	-2.16	putative hexulose 6 phosphate synthase	FIG01047465: hypothetical protein
2546	STM2765	STM14_3332	CDS		2927301	2927567	-	1.01	2.10	0.29	0.00	0.01	-2.09	putative transposase	Mobile element protein
2552	STM2772	STM14_3339	CDS	hin	2935077	2935649	+	1.18	3.19	0.12	-0.39	-0.48	-3.67	DNA-invertase Hin; DNA-invertase hin (SW:HIN_	DNA-invertase
2559	STM2779	STM14_3349	pseudo		2946329	2946862	+	1.63	2.60	0.19	0.07	0.06	-2.54	pseudogene; frameshift	
2566	STM2786	STM14_3361	CDS	tctC	2954825	2955802	+	1.67	2.88	0.15	-1.45	-2.09	-4.97	tricarboxylic transport	Tricarboxylate transport protein TctC
2589	STM2808	STM14_3390.J	CDS	nrdF	2974761	2975831	+	1.63	2.58	0.19	-1.63	-3.07	-5.66	ribonucleoside-diphosphate reductase R2-2, be	Ribonucleotide reductase of class Ib (aerobic), beta subuni
2651	STM2875	STM14_3474	CDS	hilD	3038073	3039002	+	1.25	2.84	0.16	-1.66	-1.57	-4.41	invasion protein regulatory protein; HilD (gi|44	Type III secretion transcriptional regulator HilD
2676	STM2900	STM14_3499	CDS	invH	3064626	3065069	+	1.89	4.73	0.03	-3.69	-2.86	-7.59	needle complex outer membrane lipoprotein p	Invasion protein invH precursor
2733	STM2958	STM14_3566	CDS	barA	3126329	3129085	+	2.60	4.67	0.03	0.15	0.15	-4.53	hybrid sensory histidine kinase BarA; part of th	BarA sensory histidine kinase (= VarS = GacS)
2768	STM2993	STM14_3608	CDS	recD	3164085	3165920	-	1.26	2.54	0.20	-0.31	-0.34	-2.88	exonuclease V subunit alpha; helicase/nuclease	Exodeoxyribonuclease V alpha chain (EC 3.1.11.5)
2858	STM3080	STM14_3723	CDS		3260953	3261426	-	1.62	4.40	0.04	-5.56	-4.70	-9.10	putative mannitol dehydrogenase	Putative mannitol dehydrogenase
2900	STM3124	STM14_3775.R	CDS		3302280	3303026	+	2.38	4.58	0.03	-0.07	-0.06	-4.65	Putative response regulator	Putative response regulator
2914	STM3138	STM14_3799	CDS		3318870	3319928	-	0.89	1.83	0.37	-2.11	-2.64	-4.47	putative methyl-accepting chemotaxis protein;	Putative methyl-accepting chemotaxis protein
2932	STM3156	STM14_3822	CDS		3336303	3336707	+	1.26	2.57	0.20	-1.40	-2.07	-4.64	putative cytoplasmic protein	putative cytoplasmic protein
2944	STM3168	STM14_3839	CDS	ygiR	3347190	3349361	-	1.15	2.71	0.18	-1.32	-1.55	-4.26	hypothetical protein; putative FeS oxidoreduct:	Probable Fe-S oxidoreductase family 2
2945	STM3169	STM14_3841	CDS	dctP-1 [J]_	3349849	3350832	+	0.95	2.03	0.31	-0.91	-1.44	-3.47	putative periplasmic dicarboxylate-binding prot	TRAP-type C4-dicarboxylate transport system, periplasmic
2961	STM3187	STM14_3861	CDS	ygiB	3368507	3369178	+	1.25	2.54	0.20	-0.19	-0.30	-2.84	hypothetical protein	UPF0441 protein ygiB
3046	STM3278	STM14_3960	CDS		3457380	3457910	+	1.13	1.70	0.42	-1.06	-1.14	-2.84	putative cytoplasmic protein	FIG074102: hypothetical protein
3047	STM3279	STM14_3961	CDS	mtr	3457971	3459215	-	1.21	2.43	0.22	-0.59	-0.68	-3.11	tryptophan permease; tryptophan transporter (	Tryptophan-specific transport protein
3056	STM3290	STM14_3973	CDS	argG	3472107	3473450	+	4.35	4.76	0.03	0.08	0.06	-4.70	argininosuccinate synthase; catalyzes the form:	Argininosuccinate synthase (EC 6.3.4.5)
3142	STM3388	STM14_4086	CDS	ylaB [R]	3571825	3573924	+	2.31	2.61	0.19	0.07	0.13	-2.48	putative signal transduction protein; membran(	FIG00554530: hypothetical protein
3231	STM3505	STM14_4221	CDS	feoA	3677788	3678015	+	0.82	2.31	0.25	0.13	0.17	-2.14	ferrous iron transport protein A; similar to Esch	Ferrous iron transport protein A
3232	STM3506	STM14_4222	CDS	feoB	3678034	3680352	+	1.21	2.82	0.16	-0.37	-0.51	-3.34	ferrous iron transport protein B; cytoplasmic m	Ferrous iron transport protein B
3331	STM3605	STM14_4337	CDS		3792807	3793163	+	1.16	2.21	0.27	-0.77	-0.85	-3.06	putative phage endolysin	Phage-like lysozyme
3390	STM3663	STM14_4418	CDS	bax	3863082	3863906	-	2.06	2.67	0.18	-0.69	-0.66	-3.33	hypothetical protein; similar to Escherichia coli	BAX protein
3457	STM3737	STM14_4500	CDS		3943102	3943983	+	2.79	3.43	0.09	-0.15	-0.27	-3.71	putative Zn-dependent hydrolase; similar to gly	Putative beta-lactamase
3473	STM3755	STM14_4524	CDS	rhuM	3965425	3966462	+	1.17	1.63	0.44	-1.08	-1.26	-2.89	putative cytoplasmic protein; RhuM (gi|43246C	Putative DNA-binding protein in cluster with Type I restrict
3499	STM3780	STM14_4559	CDS	gatY	3993522	3994382	-	0.91	2.06	0.30	0.29	0.46	-1.60	putative fructose-1,6-bisphosphate aldolase; cl	Fructose-bisphosphate aldolase (EC 4.1.2.13)
3572	STM3858	STM14_4653	CDS	fruA-2 [J]	4078485	4079858	-	1.86	2.89	0.15	-2.98	-2.82	-5.72	putative phosphotransferase system fructose-s	PTS system, fructose-specific IIB component (EC 2.7.1.69) /
3639	STM3942	STM14_4743	CDS		4163805	4164167	+	2.51	2.90	0.15	-0.23	-0.22	-3.12	putative cytoplasmic protein	FIG01046806: hypothetical protein
3656	STM3960	STM14_4763	CDS	rhtB	4178820	4179440	-	1.31	3.49	0.09	-0.03	-0.04	-3.53	homoserine/homoserine lactone efflux protein	Homoserine/homoserine lactone efflux protein
3733	STM4042A	STM14_4861.J	CDS		4266377	4266991	+	2.04	4.38	0.04	0.16	0.26	-4.11	putative branched-chain amino acid permease	Transcriptional regulator
3758	STM4069	STM14_4893	CDS		4293425	4293589	+	1.89	2.92	0.15	0.03	0.05	-2.87	putative periplasmic protein	Putative periplasmic protein
3787	STM14_4925.P	STM14_4925.P	sRNA_JV	IsrP [P]	4320304	4320451	+	1.01	2.46	0.22	0.63	0.40	-2.07	expressed under invasion conditions	
3896	STM4229	STM14_5085	CDS	malE	4462870	4464297	-	1.82	2.61	0.19	-0.52	-0.68	-3.29	maltose ABC transporter periplasmic protein; fı	Maltose/maltodextrin ABC transporter, substrate binding p
3916	STM4253	STM14_5111	CDS		4486168	4486623	-	1.49	2.83	0.16	-0.98	-1.82	-4.65	putative outer membrane lipoprotein	Putative outer membrane lipoprotein
3947	STM4284	STM14_5153	CDS	yjcO	4538026	4538715	-	1.21	3.06	0.13	0.21	0.22	-2.84	TPR repeat-containing protein; contains TPR re	FIG01200701: possible membrane protein
4058	STM4418	STM14_5308	CDS	IolT [R]	4672255	4673688	-	1.99	3.80	0.07	-0.51	-0.52	-4.32	sugar transporter; similar to Escherichia coli xyl	Major myo-inositol transporter IolT
4090	STM4450	STM14_5341	CDS	relE	4704513	4704797	+	3.87	6.21	0.01	-0.04	-0.06	-6.27	putative inner membrane protein	RelE/StbE replicon stabilization toxin
4113	STM4473	STM14_5369	CDS	yjgM	4727819	4728322	-	0.54	1.74	0.41	-1.04	-1.37	-3.11	putative acetyltransferase; hypothetical 18.3 kI	Aspartate N-acetyltransferase (EC 2.3.1.17)
4115	STM4475	STM14_5371	CDS	valS	4729846	4732701	-	1.08	2.75	0.17	-1.01	-1.98	-4.73	valyl-tRNA synthetase; valine--tRNA ligase; ValF	Valyl-tRNA synthetase (EC 6.1.1.9)

4142	STM4502	STM14_5403	CDS		4769156	4769764	+	0.96	2.06	0.30	-2.50	-3.98	-6.05	putative cytoplasmic protein	FIG074102: hypothetical protein
4144	cand5001	STM14_5404.P2	sRNA_cand		4771062	4771263	-	1.21	2.52	0.21	-1.80	-2.26	-4.77		
4151	STM14_5413	STM14_5413	CDS		4775969	4776121	+	0.81	1.48	0.49	-2.13	-2.76	-4.24	hypothetical protein	FIG01045355: hypothetical protein
4162	STM4519	STM14_5430	CDS	gabD-1 [J]_	4787291	4788661	+	2.90	4.07	0.05	-1.73	-1.74	-5.80	putative NAD-dependent aldehyde dehydroger	Succinate-semialdehyde dehydrogenase [NAD] (EC 1.2.1.24
4164	STM4521	STM14_5432	CDS	yjiS	4788866	4789030	+	1.62	3.10	0.13	-1.67	-1.73	-4.83	putative cytoplasmic protein	FIG074102: hypothetical protein
4248	PSLT012	STM14_5536.J2	CDS		7446	7589	-	0.90	2.67	0.18	-0.34	-0.37	-3.05	hypothetical protein	
4275	PSLT039	STM14_5562	CDS	spvB	28183	29958	-	1.83	2.78	0.17	-2.56	-4.59	-7.37	hydrophilic protein; involved in Salmonella plas	Actin-ADP-ribosyltransferase, toxin SpvB
4312	STM14_5594	STM14_5594	sRNA	finP	61461	61628	-	1.80	2.81	0.16	-0.13	-0.18	-2.99		
4334	PSLT097	STM14_5615	CDS	traF	76661	77410	+	0.81	1.48	0.49	-0.38	-0.45	-1.93	conjugative transfer: assembly	IncF plasmid conjugative transfer pilus assembly protein Tr
4340	PSLT103	STM14_5621	CDS	traT	83189	83920	+	1.37	2.77	0.17	-3.98	-3.58	-6.35	conjugative transfer: surface exclusion	IncF plasmid conjugative transfer surface exclusion protein
